# The Cytidine N-Acetyltransferase NAT10 Promotes Thalamus Hemorrhage-Induced Central Poststroke Pain by Stabilizing Fn14 Expression in Thalamic Neurons

**DOI:** 10.1007/s12035-024-04454-4

**Published:** 2024-09-13

**Authors:** Tianfeng Huang, Yang Zhang, Yan Niu, Yinggang Xiao, Yali Ge, Ju Gao

**Affiliations:** 1 Department of Anesthesiology, Northern Jiangsu People’s Hospital Affiliated to Yangzhou University, Yangzhou University, 98 Nan Tong Western Road, Yangzhou, Jiangsu 225001 P. R. China; 2 Yangzhou Key Laboratory of Anesthesiology, Northern Jiangsu People’s Hospital Affiliated to Yangzhou University, Yangzhou University, 98 Nan Tong Western Road, Yangzhou, Jiangsu 225001 P. R. China

**Keywords:** NAT10, ac4C, Hemorrhagic stroke, Thalamic pain, Central poststroke pain

## Abstract

The recognition of RNA N4-acetylcytidine (ac4C) modification as a significant type of gene regulation is growing; nevertheless, whether ac4C modification or the N-acetyltransferase 10 protein (NAT10, the only ac4C “writer” that is presently known) participates in thalamus hemorrhage (TH)-induced central poststroke pain (CPSP) is unknown. Here, we observed NAT10 was primarily located in the neuronal nuclei of the thalamus of mice, with Fn14 and p65. An increase of NAT10 mRNA and protein expression levels in the ipsilateral thalamus was observed from days 1 to 14 after TH. Inhibition of NAT10 by several different approaches attenuated Fn14 and p65 upregulation of TH mice, as well as tissue injury in the thalamus on the ipsilateral side, and the development and maintenance of contralateral nociceptive hypersensitivities. NAT10 overexpression increased Fn14 and p65 expression and elicited nociceptive hypersensitivities in naïve mice. Our findings suggest that ac4C modification and NAT10 participate in TH-induced CPSP by activating the NF-κB pathway through upregulating Fn14 in thalamic neurons. NAT10 could serve as a promising new target for CPSP treatment.

**Supplementary Information:**

The online version contains supplementary material available at 10.1007/s12035-024-04454-4.

## Introduction

Central post-stroke pain (CPSP) is a type of pain that occurs after an ischemic or hemorrhagic stroke, accompanied by abnormal sensations [1]. Spinal pain dysfunction, medial thalamic disinhibition, and neuronal hyperexcitability combined with deafferentation from thalamocortical areas are potential mechanisms of central pain and are implicated in CPSP pathogenesis [2, 3]. CPSP has a long duration and is less sensitive than peripheral neuropathy and postherpetic neuralgia to commonly used clinical first-line treatments for neuropathic pain, such as gabapentin and pregabalin [4, 5]. Because the pathogenesis of CPSP is insufficiently understood, there is still no effective way to treat CPSP [6]. Therefore, elucidating the pathogenesis of CPSP is crucial for developing reasonable clinical treatment plans.

RNA modification has been increasingly studied in the field of neuropathic pain [7, 8]. Notably, in a recent study, our group found that the expression of the RNA demethylation enzyme FTO was significantly increased in a thalamic hemorrhage (TH) CPSP model and that FTO performs a crucial function in thalamic pain by modulating the m6A modification of neuronal Tlr4 mRNA [9]. To additionally assess the functional role of epigenetic mRNA modifications in CPSP, we focused our research on the newly discovered N4-acetylcytidine (ac4C) modification. This modification, reported by Arongo et al. in 2018 [10], increases mRNA stability and translation rate.

The ac4C modification is catalyzed by N-acetyltransferase 10 (NAT10), the only reported mammalian RNA ac4C “writer” protein. NAT10 is expressed primarily in the nucleolus of cells and is involved in the regulation of telomerase activity, rRNA transcription, and cytokinesis [11, 12]. Arango et al. [10] illustrated the modification of ac4C in human HeLa cell mRNA and showed the NAT10 knockout cell line exhibited a substantial decrease in the level of acylated mRNAs. Laguna et al. [13] reported a significant reduction in the plasma ac4C concentration in patients with pulmonary fibrosis in comparison to the normal level. In addition, ac4C modification of long noncoding RNAs may be linked to the onset and advancement of Alzheimer’s disease, and numerous studies have documented the involvement of NAT10 in various biological processes, like depression and senescence [14, 15]. However, the roles of NAT10 and ac4C modification in CPSP are still poorly understood.

In this study, for the first time, we combined multiple sequencing technologies with several NAT10 inhibition methods to determine the roles of NAT10 and ac4C in CPSP. We found that the increase in fibroblast growth factor-inducible-14 (Fn14) protein expression and the nuclear factor kappa B (NF-κB) pathway activation could be blocked by the knockdown of Nat10 or treatment with Remodelin (a NAT10 inhibitor). According to the results, NAT10 was upregulated to initiate and maintain TH-induced CPSP, since the upregulation of NAT10 activates the NF-κB pathway by upregulating Fn14. Thus, the epigenetic mechanism involving NAT10-mediated ac4C RNA modification is suggested to have an essential function in TH-induced pain hypersensitivity, a discovery that might lead to a novel perspective on the processes behind CPSP and point to the potential role of NAT10 as a target for the development of CPSP treatment strategies.

## Materials and Methods

The Medical College of Yangzhou University’s Animal Care and Use approved this study, and it followed all the guidelines set forth by the International Association for Pain Research.

### Animals

We purchased C57BL/6 male mice (around 7 to 8 weeks of age) from the Comparative Medical Center of Yangzhou University and Beijing Weitong Lihua Experimental Animal Technical Co., Ltd. Nat10-flox mice (strain no. T007971) and Calb1-P2A-iCre mice (strain no. T006202) were procured from GemPharmatech (Nanjing, China). According to the structure of Nat10 gene, exon4-exon5 of Nat10-201(ENSMUST00000028608.12) transcript is recommended as the knockout region. The two strains were mated to produce mice with a loss of Nat10 function in the brain’s neurons. A previous study showed that NAT10 is critical for mouse development and that homozygous knockout of NAT10 causes embryonic lethality [16]. Indeed, we did not obtain healthy homozygous knockout mice during the construction of the NAT10 knockout mouse model; therefore, we used heterozygous Nat10^+/−^ mice screened by PCR genotyping in this study. All heterozygotes did not display any observable differences in phenotype, such as size, coat color, or behavior. All mice were kept in captivity in animal facilities on a standard light-and-dark cycle running for 12 h with an unlimited supply of food pellets and standard laboratory water.

### TH Model

Mice were anesthetized with evaporated isoflurane (5% induction, 2% maintenance) before being fixed on a stereotaxic frame (Kopf Instruments). Subsequently, under stereotactic guidance as previously described [9, 17] Collagenase (Coll) IV (Sigma-Aldrich Co., St. Louis, MO; 0.01 U/10 nL, saline solution-dissolved) or autologous blood [18] (15 μL, extracted via the tail vein) was administered via microinjection into the right ventral posteromedial nucleus (VPM) and ventral posterolateral nucleus (VPL) of the thalamus (anterior–posterior-anterior-fontanel-0.82–2.30 mm: posterior 1.30–1.95 mm on the lateral side of the midline, and 3.01–4.25 mm on the surface of the skull’s ventral side). In addition, the sham-operated group was administered an injection of an equivalent volume of sterile saline. After the treatment, the glass micropipette remained in this orientation for 10 min to facilitate the complete dispersion of the Coll IV or autologous blood.

### Behavioral Tests

Assessments of pain behavior, encompassing responses to cold, thermal, and mechanical stimuli, were performed at one-hour intervals. To minimize variation among and within individuals during the assessment of behavioral outcomes, the animals underwent training for 1–2 days before the commencement of behavioral assessment. The experimentalist remained blinded to the treatment parameters during the behavioral tests.

First, the measurement of the paw withdrawal frequency following mechanical stimulation was conducted as previously documented [17, 19]. Briefly, the mice were placed alone in a Plexiglass chamber on an elevated screen and allowed to adapt for 30 min. The rear paw was stimulated for 1–2 s utilizing two calibrated von Frey filaments (0.07 and 0.4 g; Stoelting Co.). This procedure was replicated ten times, with a 5-min interval between each rear paw stimulation. Positive responses were characterized by swift withdrawal of the paws. Percentage calculations were conducted to determine the frequency of paw withdrawal responses to each of the ten stimuli: [(number of paw withdrawals/10 trials) × 100% = response frequency].

Then, IITC, Inc.; Life Science Instruments, Woodland Hills, CA supplied the model 336 analgesic meter, which was subsequently utilized to measure the paw retraction latency in reaction to harmful heat stimuli, as previously detailed [9]. Briefly, a glass plate was utilized to set up the mice in a Plexiglas enclosure. The center of the sole surface of every rear paw was illuminated by a beam emanating from a lightbox. Then, the light was switched off in response to a swift upward movement of the hind paw. Paw latency was a term used to denote the duration of illumination. Five experiments were performed for each side, with a 5-min interval between each experiment. To prevent tissue damage, we established a cutoff time of 20 s.

Finally, as was earlier reported, the paw withdrawal latency was assessed by employing a cold aluminum plate in the presence of hazardous cold (0 ℃) [17]. Briefly, every mouse was affixed to a Plexiglas chamber positioned atop a level plate, and the internal temperature was tracked consistently with a thermometer. A “paw jump delay” was established to denote the duration between mouse placement and jumping. Each experiment was repeated three times at 10-min intervals. Tissue damage was prevented by employing a 20-s cutoff time.

As stated previously [20], motor function tests were conducted after the completion of the pain behavioral tests. These tests encompassed assessments of placement, gripping, and righting reflexes. To assess placement reflexes, the rear limbs were positioned marginally below the forelimbs, with the hind paws’ backs in contact with the table’s edge. Subsequently, the motion of the rear paw on the tabletop was recorded. Then, the animal’s grasping reflexes were evaluated by placing it on a wire grille and recording whether or not the back paw grasped the wire. The righting reflex was evaluated by placing the animal on its back on a level surface and observing if it could return to a normal upright posture without delay. At 5-min intervals, every test was replicated five times, and the results were documented through the computation of the count of normal reflexes shown per test.

### Single-Nucleus RNA Sequencing (snRNA-seq)

The snRNA-seq dataset originated from our prior experimental studies, consisting of four distinct samples: a pair identified as the Control group (samples C1 and C2) and another pair representing the Model group (samples M1 and M2). The snRNA data were reported previously and analyzed again. The pertinent data has been deposited into the Gene Expression Omnibus repository under the accession number GSE227033 [21]. CellRanger was subsequently applied to quantify cells based on barcodes, and the average numbers of reads and genes per cell in each sample were counted. Afterwards, Seurat software (version 4.0.5) (parameters: min.cells = 3, min.features = 200) was chosen to filter out low-quality cells [22]. The following screening criteria were used: a library size greater than the median + 2 times the median absolute deviation (MAD), a gene count greater than the median ± 2 times the MAD, and a mitochondrial body content greater than 10%. The data were standardized, homogenized, subjected to principal component analysis and uniform manifold approximation and projection analysis, and processed in sequence. The optimal number of principal components was determined through ElbowPlot. Clusters were annotated based on cell types that are important for the occurrence of disease through the Celldex package [23].

### Acetylated RNA-Binding Protein Immunoprecipitation (acRIP) and acRIP Sequencing (acRIP-seq)

Guangzhou Epibiotek Co., Ltd performed acRIP and acRIP-seq. Total RNA was extracted using TRIzol™ Reagent (Invitrogen, 15596018). The concentration of total RNA was measured by Qubit RNA HS assay kit (Invitrogen, Q32852). One hundred micrograms of total RNA was fragmented into 100–200 nt RNA fragments using 10X RNA Fragmentation Buffer (100 mM Tris–HCl, 100 mM ZnCl2 in nuclease-free H2O). The reaction was stopped by adding 10X EDTA (0.5 M EDTA). Acetylated RNA immunoprecipitation was performed using EpiTM ac4C immunoprecipitation kit (Epibiotek, R1815). Briefly, the fragmented RNA was incubated with Anti-N4-acetylcytidine (ac4C) antibody (abcam, ab252215) for 3 h at 4 ℃ and then with protein A/G magnetic beads (Thermo Fisher Scientific, 88802) at 4 ℃ for an additional 2 h to obtain immunoprecipitated RNA fragments. The ac4C-enriched RNA was purified by Zymo RNA clean and concentrator-25 kit (Zymo Research, R1017). The library was prepared by EpiTM mini longRNA-seq kit (Epibiotek, E1802). Both the input samples without IP and the ac4C IP samples were subjected to 150-bp, paired-end sequencing on an Illumina NovaSeq 6000 sequencer.

Adapters were trimmed and sequences were filtered utilizing Cutadapt (v2.5). Subsequently, the HISAT2 aligner (version 2.1.0) was utilized to align the remainder of the reads with the human Ensembl genome GRCh38 (mouse Ensembl genome GRCm38). Differential ac4C peaks were identified using the exomePeak R package. Genomic characteristics associated with ac4C-RNA were represented graphically utilizing the Guitar R package (v1.16.0). We utilized HOMER (v4.10.4) with the parameters “-len 12-rna” to conduct de novo motif analysis on the identified ac4C peaks that had a *P* value < 0.05.

We utilized featureCounts (v1.6.3) to determine the genome-mapped reads. The DESeq2 R package was employed to execute the differential expression of gene (DEG) analysis. The clusterProfiler R package (v3.6.0) was adopted for Gene Ontology (GO) and Kyoto Encyclopedia of Genes and Genomes (KEGG) analyses.

### Ribosome Profiling Sequencing (Ribo-seq) and RNA Sequencing (RNA-seq)

Guangzhou Epibiotek Co., Ltd conducted the RNA-seq and Ribo-seq. For Ribo-seq, a total of 107 cells were prepared for cell lysis. Then, the cell medium was discarded, and the cells were washed twice with ice-cold PBS containing 100 μg/mL cycloheximide (Adooq, 66–81-9). 100 mg tissue were pulverized manually under liquid nitrogen and treated by ice-cold PBS containing 100 μg/mL cycloheximide. Ribosome profiling was performed using Epi™ Ribosome Profiling Kit (Epibiotek, R1814). Subsquently, RPFs (ribosome-protected RNA fragments) was extracted using RNA clean&ConcentratorTM-5 kit (ZYMO, R1016). EpiTM RiboRNA Depletion Kit (Human/Mouse/Rat) (Epibiotek, R1805) was used for rRNA depletion. Sequencing libraries were constructed using QIAseq miRNA Library kit (QIAGEN, 1,103,679). The revised riboWaltz package was utilized to estimate the codon usage frequency and trinucleotide periodicity of ribosomes. The Price software was utilized to identify ORFs. The featureCounts program was used to determine the number of reads. The DESeq2 package’s Fragments Per Kilobase of transcript per Million fragments mapped function was utilized to additionally normalize the raw counts to the Reads Per Kilobase of transcript per Million reads mapped values. The translational efficiency was calculated by comparing the normalized abundance found by RNA-seq to that found by ribosome profiling. The clusterProfiler package in R software was adopted to perform GO and KEGG analyses.

For RNA-seq, a library was established with a VAHTS Stranded mRNA-seq Library Prep Kit for Illumina V2 (Vazyme Biotech, NR612-02) as per the directions stipulated by the manufacturer. The HISAT2 aligner (v2.1.0) was employed with the parameters as follows: “–rna-strandness RF” to align the reads to the human Ensemble genome GRCh38 (mouse Ensemble genome GRCm38). We utilized featureCounts (v1.6.3) to determine the genome-mapped reads. The DESeq2 R package was used to conduct the DEG analysis. The clusterProfiler package in R (v3.6.0) was utilized to execute GO and KEGG analyses.

### In Vivo AAV Infection

The virus was microinjected as described for Coll IV. For experiments in which NAT10 was knocked down, C57BL/6 mice were microinjected into the thalamus with a single dosage of 2 × 10^11^ vector genomes of adeno-associated virus (AAV) serotype 9 containing either a NAT10-interfering sequence (AAV9-sh-NAT10) or control-interfering sequence (AAV9-sh-Con) with the CMV promoter. For experiments in which NAT10 was overexpressed, C57BL/6 mice were microinjected into the thalamus with a single dosage of 2 × 10^11^ vector genomes of AAV serotype 5 containing either NAT10 (AAV5-NAT10) or the EGFP control gene (AAV5-EGFP) with the CMV promoter. OBiO Technology (Shanghai) Corp., Ltd. constructed and supplied the AAV9-sh-Con, AAV9-sh-NAT10 (contract number: HYKY-230323052-DAAV), AAV5-EGFP, and AAV5-NAT10 (contract number: HYKY-220330045-DAAV). The sequences of shRNA and control are shown in Supplementary Table 1.

### Nissl Staining

Isoflurane was used to induce anesthesia in mice, and 50–100 ml of 4% paraformaldehyde in 0.1 M phosphate-buffered saline (PBS, pH 7.4) was perfused into these mice. After collecting the brains and postfixing them at 4 °C for a whole night, cryoprotection was performed for two days in 0.1 M PBS consisting of 30% sucrose. Following sectioning the brains using a cryostat microtome (Leica) to an approximate thickness of 30 μm, Nissl staining was performed with cresyl violet, as previously documented [9, 17, 20]. The NIH ImageJ program was utilized to analyze the optical microscopy (Leica DMI4000) images to ascertain the location and sizes of the damaged regions.

### ac4C Dot Blot Assay

One microgram of RNA was subjected to denaturation at 75 °C for 5 min, followed by 1 min of immediate ice storage, loading onto Hybond-N + membranes, and another 10 min of crosslinking with UV254. Following a 2-h blocking process with 5% nonfat milk at room temperature, the membranes were subjected to incubation at 4 °C through the night with an antibody that is specific for ac4C (Abcam; ab252215; 1:1000). The next step involved washing the membrane thrice in PBS with Tween20 (PBST), probing them for 1 h at room temperature with HRP-conjugated secondary anti-rabbit IgG in PBST and washing them again in PBST. The enhanced chemiluminescence (ECL) (34,577, Thermo Scientific) was employed to observe the signal. The membranes were stained with 0.2% methylene blue to verify Equal RNA loading. Each dot’s intensity was normalized to the quantity of total RNA.

### Total RNA Isolation and Quantitative Real-Time PCR

We followed our earlier published protocol for extracting RNA and performing RT-qPCR [9, 17]. Briefly, mice were decapitated, and the VPL/VPM were obtained rapidly after which total RNA was isolated with a kit (Qiagen). Oligo (dT) primers and ThermoScript reverse transcriptase (Invitrogen) were subsequently utilized to reverse-transcribe the RNA. Forward and reverse primers were utilized to amplify the cDNA in triplicate within a 20 μL reaction volume [the sequences of the NAT10 PCR primers include the following: 5′-TCGAGGAAAAGATCAGGTGGT-3′ (forward) and 5′-TTCTTCTGTAGCTGCCGCAT-3′ (reverse)]. The sequences of β-actin PCR primers included the following: 5′-CCTAGGCACCAGGGTGTGAT-3′ (forward) and 5′-AGCACAGGGTGCTCCTCA-3′ (reverse) and SYBR Green Supermix (Bio-Rad). Following a 3-min incubation at 95 °C, all real-time PCR assays were conducted with 40 cycles consisting of 10 s at 95 °C, 30 s at 60 °C, and 30 s at 72 °C. The process of determining relative mRNA levels was carried out utilizing the ΔCt method (2^−ΔΔCt^). The internal control β-actin was used to normalize all the data.

### Western Blotting

We followed the steps outlined in our previous publication [9, 17] while performing the Western blotting. Briefly, the protein of thalamic homogenates was extracted following the instructions of a whole protein extraction kit (EX1100, Solarbio) and a nuclear protein extraction kit (EX1470, Solarbio). After the protein concentration was determined, 5 min of heating at 99 °C was applied to the samples. We loaded sample including 30 μg protein on the 4–20% precast PAGE gels (Genscript) per lane and then fractionated proteins. Wet transfer of the proteins to a polyvinylidene fluoride transfer membrane was performed. Following a 1-h blocking of the membrane with 5% skim milk in Tris-buffered saline with 0.1% Tween-20, the primary antibodies were introduced and left to incubate for the night at 4 °C. The membranes were rinsed using TBST and then probed at room temperature for 2 h with the secondary antibody. To visualize the proteins, Western peroxidase reagent and Clarity Western ECL Substrate (Bio-Rad) with the ChemiDoc XRS system (Bio-Rad) and Image Lab software were utilized. Quantitative data intensity was determined via optical density measurement using NIH ImageJ software. Details of the antibodies are given in Supplementary Table 2. GAPDH, β-actin and histone H3 were used as internal controls. H3 was used for proteins derived from the nucleus, such as NAT10 and nuclear p65 (N-p65). Beta-actin and GAPDH were used for proteins derived from the whole cell, such as Fn14 and total p65 (T-p65).

### Immunofluorescence

Following the administration of isoflurane to induce deep anesthesia in the mice, 50–100 mL of 4% paraformaldehyde in 0.1 M PBS (pH 7.4) was used to perfuse them. The next step involved harvesting and postfixing the brains for 24 h at 4 °C, and cryoprotecting them in 30% sucrose throughout the night. A cryostat was employed to section the tissues at a size of 30 µm in thickness. Following a 1-h blocking process with PBS that contained 5% goat serum and 0.3% Triton X-100 at 37 ℃, the samples were then treated with primary antibodies throughout the night at 4 ℃. These samples were subsequently subjected to incubation for 1 h at room temperature with secondary antibodies. Control experiments included omission of the primary antiserum and the substitution of normal mouse or rabbit serum for the primary antiserum. The Vectashield plus 4’,6-diamidino-2-phenylindole mounting medium (Vector Laboratories) or Vectamount permanent mounting medium (Vector Laboratories, Burlingame, CA) were utilized to mount the sections. Every image was acquired with a Leica DMI4000 fluorescence microscope coupled with a DFC365FX camera (Leica, Germany). Lastly, the cells that were labelled as single, double, or triple were tallied manually or with the NIH ImageJ program. Antibody-specific information is provided in Supplementary Table 3.

### Statistical Analysis

Mice were classified at random into different treatment groups. Each result is given as the mean ± SD. Both one-way and two-way ANOVAs were implemented for data analysis. After identifying significant variations through ANOVA, pairwise comparisons of the means were made via the post-Tukey method (SigmaPlot 12.5, San Jose, CA). The criterion for significance was established at *P* < 0.05.

## Results

### NAT10 Is Significantly Upregulated After TH and Is Mainly Expressed in Neurons

The CPSP model induced by Coll IV was established to determine the roles of ac4C modification and NAT10 more specifically in TH-induced central pain. Compared to control mice, model mice with TH exhibited aberrant cold pain, hyperalgesia, and severe and prolonged aberrant mechanical pain on the contralateral side, which was sustained for a minimum of 14 days (Supplementary Fig. 1).

**Fig. 1:**
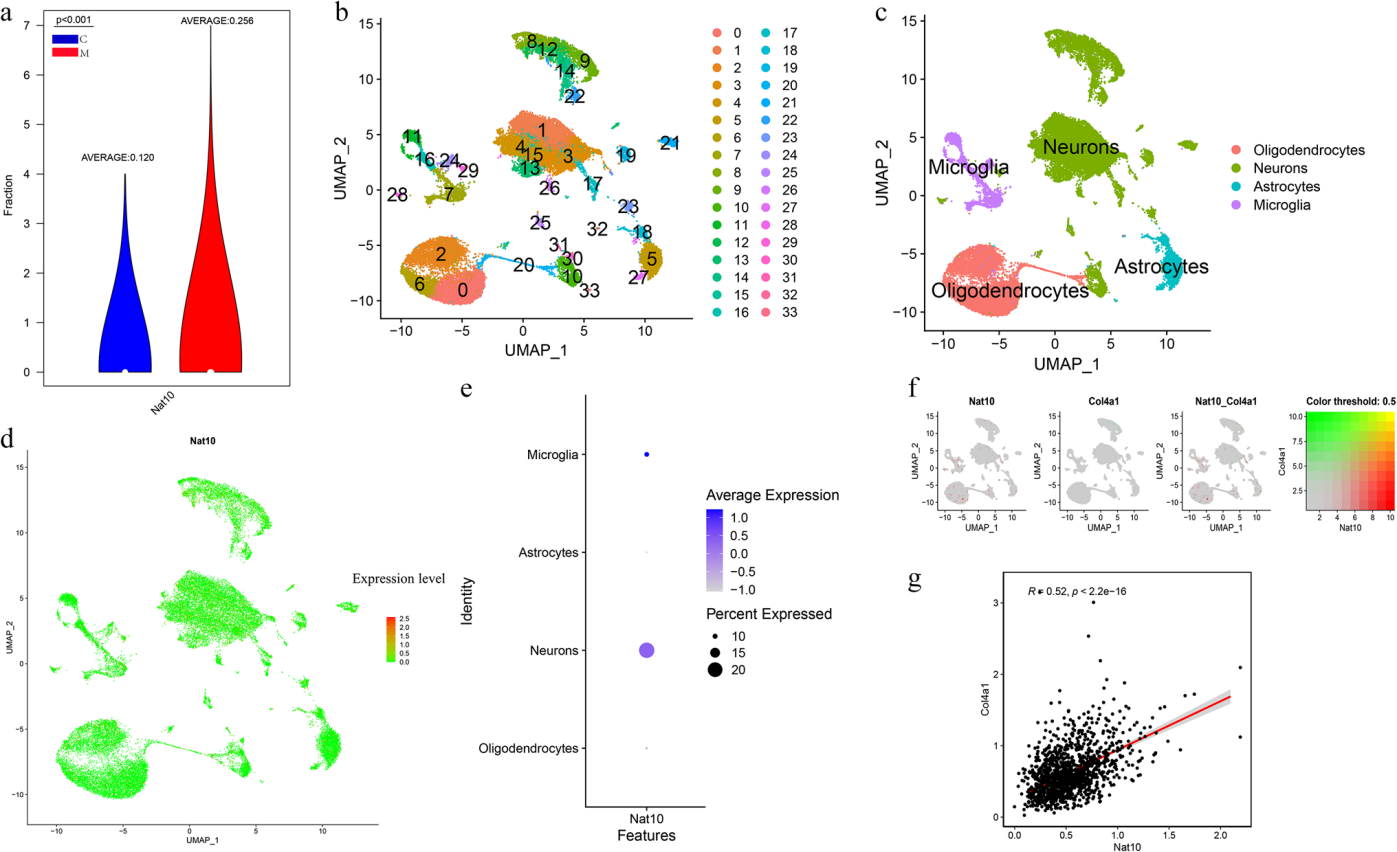
snRNA-seq data showing the expression and location of NAT10. NAT10 was significantly increased in the model group, n of C = 20,119, n of M = 12,213, independent samples *t*-test with false discovery rate (**a**). A total of 34 subgroups were obtained through uniform manifold approximation and projection analysis (**b**). The thirty-four clusters were assigned to the following four cell types: oligodendrocytes, neurons, astrocytes, and microglia (**c**). A scatter plot (**d**) and bubble plot (**e**) show that NAT10 is expressed mainly in neurons. Colors ranging from light to dark represent the expression from low to high, the sizes of circle represent the percentage of cells expressing NAT10 among all corresponding cells (**d**, **e**). NAT10 and Col4a1 are coexpressed mainly in neurons, the table on the far left showed that the relative gene expression levels represented by each color (**f**), and the Pearson correlation analysis showed that NAT10f is closely related to the disease progression gene Col4a1 (**g**). M, model group; C, control group

After successfully establishing the TH model mice, we sought to explore the expression of Nat10 in TH based on snRNA-seq data. The TH group exhibited a remarkable upregulation of Nat10 (Fig. 1a). For this study, we utilized the R package SingleR to annotate each cluster. Out of the 34 clusters, we identified four different types of cells: oligodendrocytes, neurons, astrocytes, and microglia (Fig. 1b and c). Scatter plots showed that the red spot, representing Nat10, was mainly expressed in the area representing neurons (Fig. 1d), and bubble plots showed consistent results (Fig. 1e). We visualized the coexpression of the Nat10 gene and the Col4a1, which are neuron marker genes elevated during CPSP progression. The results showed that NAT10 and Col4a1 were coexpressed mainly in neurons (Fig. 1f) and that NAT10 and Col4a1 expression levels were closely related (Fig. 1 g). Taken together, these results demonstrated that NAT10 was remarkably elevated in neurons in TH.

### RNA ac4C Modification Is Critically Involved in the Pathophysiology of TH

**Fig. 2:**
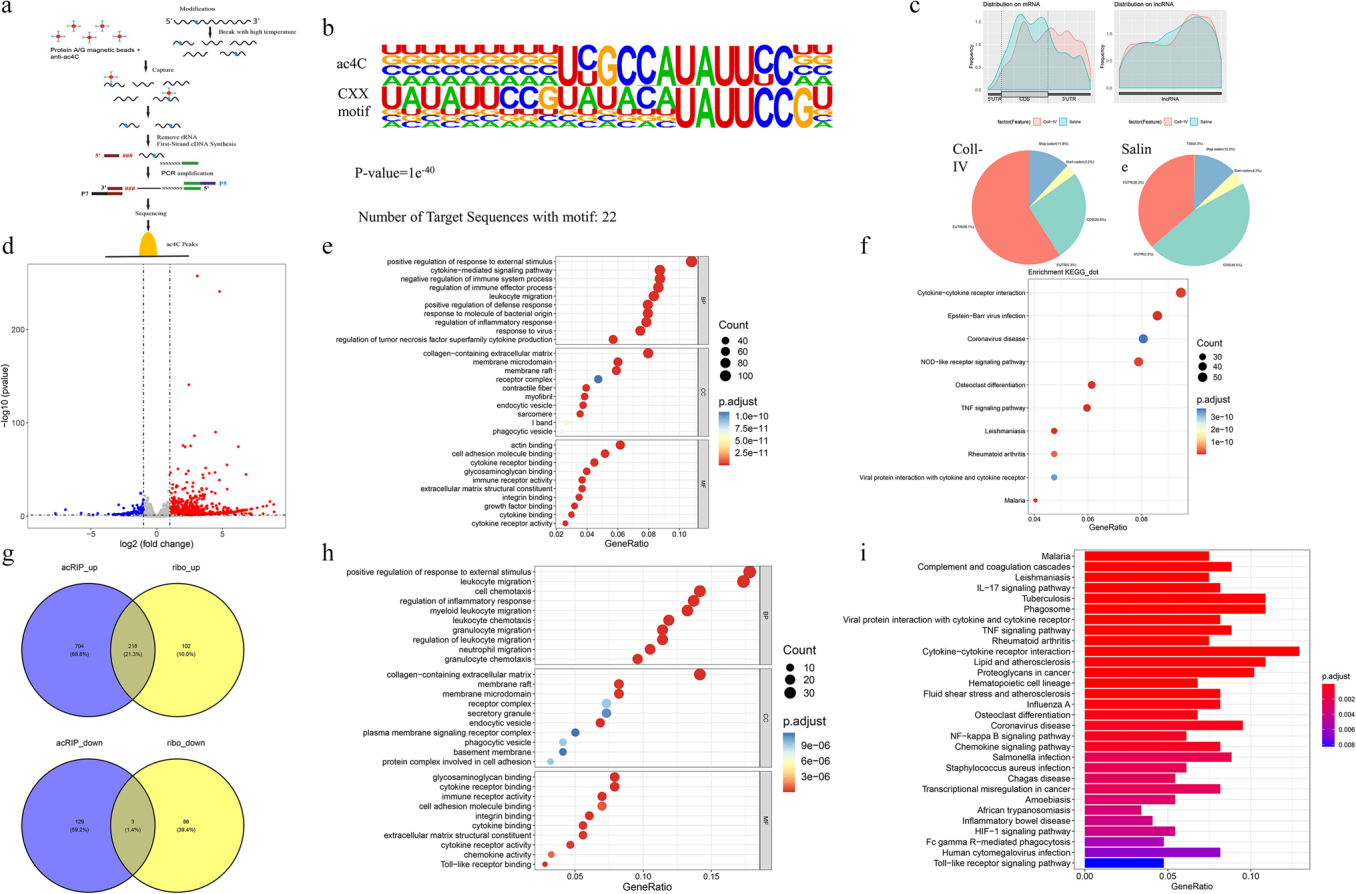
The ac4C RNA modification performs a critical function in the pathophysiology of thalamic hemorrhage. **a** Schematic of the acRIP-seq procedure. **b** Sequence logo. The up represents the ac4C motif, and the down represents the CXX motif. The size of each letter is proportional to the frequency of the corresponding base at that position. **c** The area under the curve represents the distribution of ac4C on RNA, and the two pie charts below show the percentage of ac4C in various regions of the two groups of mRNA. **d** Compared to Saline group, A volcano diagram illustrating the mRNA acetylation of genes that are upregulated (red) or downregulated (blue) in the thalamic hemorrhage model. **e** GO and **f** KEGG analysis of the differentially ac4C-expressed genes from acRIP-seq data. **g** Venn diagrams illustrate two pairs of common up-and-down-regulated genes in acRIP-seq and Ribo-seq. **h** GO and **i** KEGG analysis of the common genes whose expression is altered in the two pairs of data

acRIP-seq is a method that enriches RNA fragments with ac4C modifications by immunoprecipitating RNA modified with ac4C using a specific antibody [24]. These fragments were then sequenced and analyzed. Through acRIP-seq, we identified and quantify RNA targeted by NAT10, and by bioinformatics analysis, we screened for key downstream molecules regulated by NAT10 (Fig. 2a). The ac4C peaks contained the CXX motif, at which ac4C modification occurs (Fig. 2b), indicating the good quality of the acRIP-seq data. Notably, ac4C peaks were most commonly found in the thalamus within 3′untranslated regions (3′UTRs) and coding sequences (CDSs) (Fig. 2c), indicating that NAT10 may directly regulate mRNA expression. As opposed to the control group, the mRNA acetylation of many genes upregulated in the TH model (Fig. 2d).

The cytokine-mediated signalling pathway, cytokine binding, and cytokine receptor activity were all linked to mRNA acetylation, according to GO analysis (Fig. 2e). According to KEGG analysis, mRNA acetylation was linked to the interaction between cytokines and their receptors, the NOD-like receptor, and the TNF signalling pathways (Fig. 2f). Ribo-seq is a high-throughput sequencing technology that captures and sequences mRNA fragments bound to ribosomes, thereby providing dynamic information on protein synthesis, encompassing the efficiency and rate of gene translation [25]. We conducted a combination of acRIP-seq and Ribo-seq to delve deeper into the link between mRNA acetylation and translation in CPSP. Our research revealed that a total of 218 genes underwent ac4C modification of the corresponding mRNA, and their translation efficiency increased (Fig. 2g). GO analysis demonstrated significant enrichment of these genes primarily in the positive regulation of the response to external stimuli, regulation of the inflammatory response, and regulation of the collagen-containing extracellular matrix (Fig. 2h). KEGG analysis exhibited a significant association of these genes with the NF-κB, TNF, and the Toll-like receptor signalling pathways (Fig. 2i). Overall, these results demonstrated that RNA ac4C modification, mediated by NAT10, modulated mRNA expression and translation efficiency in TH.

### The Level of ac4C and the Expression of NAT10 at the Site of Hemorrhage Injury Were Significantly Increased on Days 1–14 After TH

**Fig. 3:**
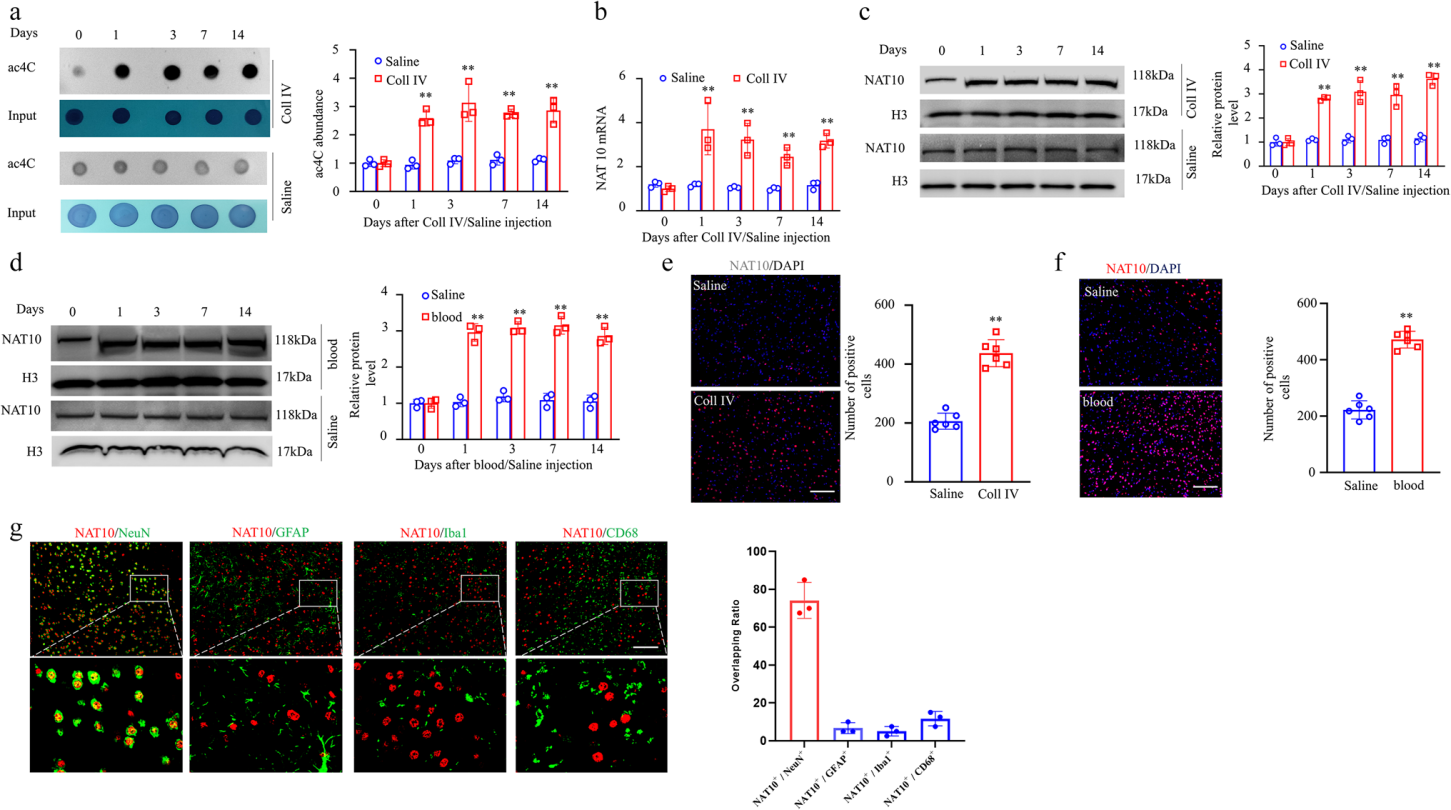
Thalamic NAT10 levels were elevated in models of thalamic pain induced by autologous blood and collagenase IV. **a** Thalamic hemorrhage increased mRNA acetylation, as shown by dot blot analysis. **b** NAT10 mRNA level in the ipsilateral (Ipsi) thalamus on various days post-treatment with saline or Coll IV. **c** NAT10 protein concentrations in the Ipsi thalamus on various days post-treatment with saline or Coll IV. **d** Variations in NAT10 protein levels within the Ipsi thalamus on distinct days following autologous whole blood or saline microinjection treatment. *n* = 3 biological repeats/group/time point. **P < 0.01 versus the corresponding control group (day 0) by two-way ANOVA followed by post hoc Tukey’s test. **e** The Ipsi thalamus was stained with NAT10 immunofluorescence on day seven following Coll IV or saline treatment. **f** The Ipsi thalamus was stained with NAT10 immunofluorescence on day seven post-microinjection of autologous whole blood or saline. *n* = 3 biological repeats/group. **P < 0.01 versus the saline group by 2-tailed unpaired Student’s *t* test. Scale bar 50 μm. **g** The representative image and overlapping ratios of NAT10 colocalized with NeuN, Iba1, GFAP, and CD68 in the Ipsi thalamus on day 7 post-microinjection of Coll IV. Representative images from 3 biological repeats (*n* = 3 mice). Scale bar 50 μm

We began by assessing whether the ac4C level in the thalamus was altered after TH-induced CPSP. Compared with the saline group, TH via Coll IV microinjection induced a time-dependent increase in ac4C levels in the thalamus (Fig. 3a). Moreover, in the Coll IV-induced TH model, NAT10 mRNA and protein expression levels continued to increase on days 1–14 after TH, relative to the saline group (Fig. 3b and c). Consistent with the results in the Coll IV-induced TH model, the NAT10 protein was also upregulated on days 1–14 after the induction of TH by microinjecting the animals with autologous whole blood (Fig. 3d). Consistently, on day 7, the density of Nat10 immunoreactivity in the thalamus was 2.12 times higher in the group that received Coll IV microinjection as opposed to the one that received saline microinjection (Fig. 3e). The results were similar on day 7 post-microinjection with autologous whole blood into the VPL and VPM of the thalamus (Fig. 3f). Most of the NAT10 immunoreactivity in thalamic cells 7 days following Coll IV treatment was found to overlap with NeuN immunoreactivity, a neuronal marker, according to a double labelling assay (Fig. 3g). These findings indicate that excess NAT10 was expressed primarily in thalamic neurons.

### Pharmacological Suppression of NAT10 Alleviates TH-Induced CPSP

**Fig. 4:**
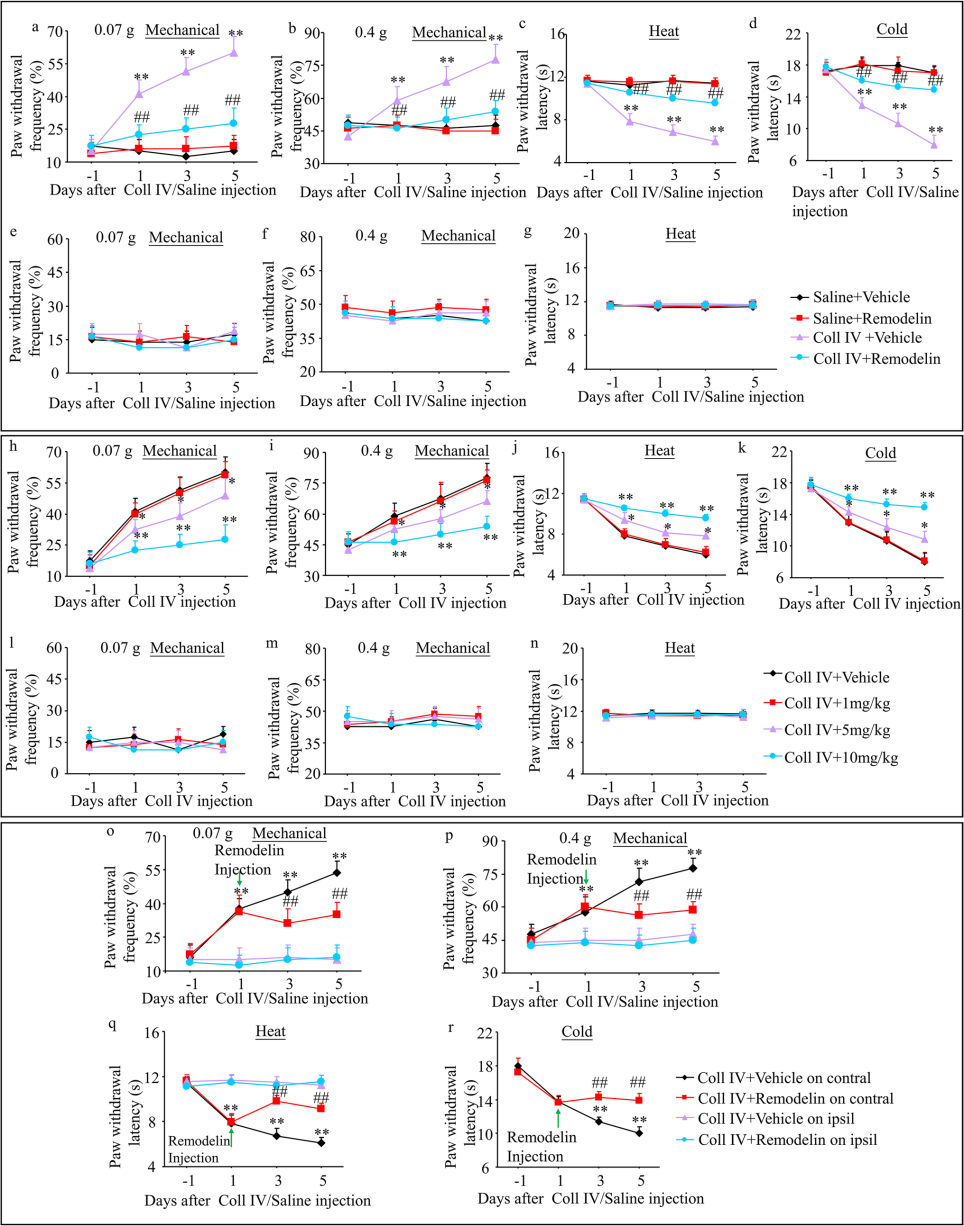
Impact of Remodelin administration via the systemic tail vein on the development of thalamic pain caused by Coll IV microinjections. **a**–**g** The animals were given either a vehicle or Remodelin (10 mg/kg) half an hour before the Coll IV or saline microinjection, and then once every day afterwards. Analysis of the impact of systemic pretreatment with Remodelin or vehicle on the frequency of paw withdrawals to 0.07 g (**a** and **e**) and 0.4 g (**b** and **f**) von Frey filaments and the latencies of paw withdrawal in response to heat (**c** and **g**) and cold (**d**) stimuli on days 1 and 5 after following Coll IV or saline microinjection on the contralateral (Contral (**a**–**d**) and Ipsi (**e**–**g**)) thalamus. **P < 0.01 versus the corresponding baseline (day -1). ##*P* < 0.01 versus the Coll IV plus vehicle group at the corresponding days. **h**–**n** Analysis of the impact of systemic pretreatment with Remodelin (0 (vehicle), 1, 5, or 10 mg/kg) on the frequency of paw withdrawals to 0.07 g (**h** and **l**) and 0.4 g (**i** and **m**) von Frey filaments and the latencies of paw withdrawal in response to heat (**j** and **n**) and cold (**k**) stimuli on days 1 and 5 post-microinjection of Coll IV on the Contra (**h**–**k**) and Ipsi (**l**–**n**) thalamus. *P < 0.05 versus the Coll IV plus vehicle group at the corresponding days. **P < 0.01 versus the Coll IV plus vehicle group at the corresponding days. **o**–**r** One day following Coll IV microinjection, patients were given either vehicle or Remodelin (10 mg/kg) once per day for 5 days. Analysis of the impact of systemic pretreatment with Remodelin or vehicle on the frequency of paw withdrawals to 0.07 g (**o**) and 0.4 g (**p**) von Frey filaments and the latencies of paw withdrawal in response to heat (**q**) and cold (**r**) stimuli on days 3 and 5 following treatment with Coll IV on the Contra and Ipsi thalamus. **P < 0.01 versus the corresponding baseline (day -1). ##*P* < 0.01 versus the Coll IV plus vehicle group at the corresponding days. **a**–**r** *n* = 8 mice/group; two-way ANOVA with repeated measures followed by post hoc Tukey’s test

We then examined whether increased thalamic NAT10 was involved in the genesis of TH-induced thalamic pain by Remodelin, a specific inhibitor of NAT10. Remodelin was given to the C57BL/6 mice via tail injection half an hour before being microinjected with Coll IV or saline and then continuously administered for 5 days. Contralateral pain hypersensitivity of these types was considerably reduced in mice microinjected with Coll IV and administered 10 mg/kg Remodelin systemically (Fig. 4a–d). A dose-dependent effect was observed (Fig. 4h–k). Additionally, as predicted, these doses of Remodelin exhibited no influence on the basal paw withdrawal frequency or latency on the ipsilateral side in the Remodelin plus Coll IV-treated group throughout the observational period (Fig. 4e–g and l–n). Furthermore, systemic Remodelin administration had no discernible impact on locomotor function, as anticipated (Supplementary Table 4).

Additionally, the function of thalamic NAT10 in the maintenance of thalamic pain caused by TH was investigated. Vehicle and Remodelin (10 mg/kg) were injected via the tail vein 1 day following treatment with Coll IV and once every day for 5 days. Compared with the ipsilateral side, systemic postadministration of Remodelin at the same dose significantly decreased mechanical allodynia, heat hyperalgesia, and cold hyperalgesia on days 3 and 5 after Coll IV microinjection on the contralateral side (Fig. 4o–r).

Additionally, we investigated if the injury to the thalamus caused by Coll IV treatment could be ameliorated by systemic injections of NAT10. Brain samples were obtained for Nissl staining following behavioral assessments. Compared with those in the Coll IV + vehicle–treatment group, the Nissl-stained cell count in the ipsilateral VPM and VPL was significantly greater in the Coll IV plus NAT10 treatment group (Supplementary Fig. 2a and b). As anticipated, the treated groups did not exhibit significant variations in the Nissl-stained cell count in the contralateral VPL or VPM (Supplementary Fig. 3a and b).

Similar to Coll IV-induced TH, the behavioral response was observed post-injection of Remodelin systemically into the tail vein in a model of thalamic pain induced by autologous whole-blood microinjection (Supplementary Fig. 4 and Table 4).

### Genetic Knockdown of Thalamic NAT10 Attenuates TH-Induced CPSP

**Fig. 5:**
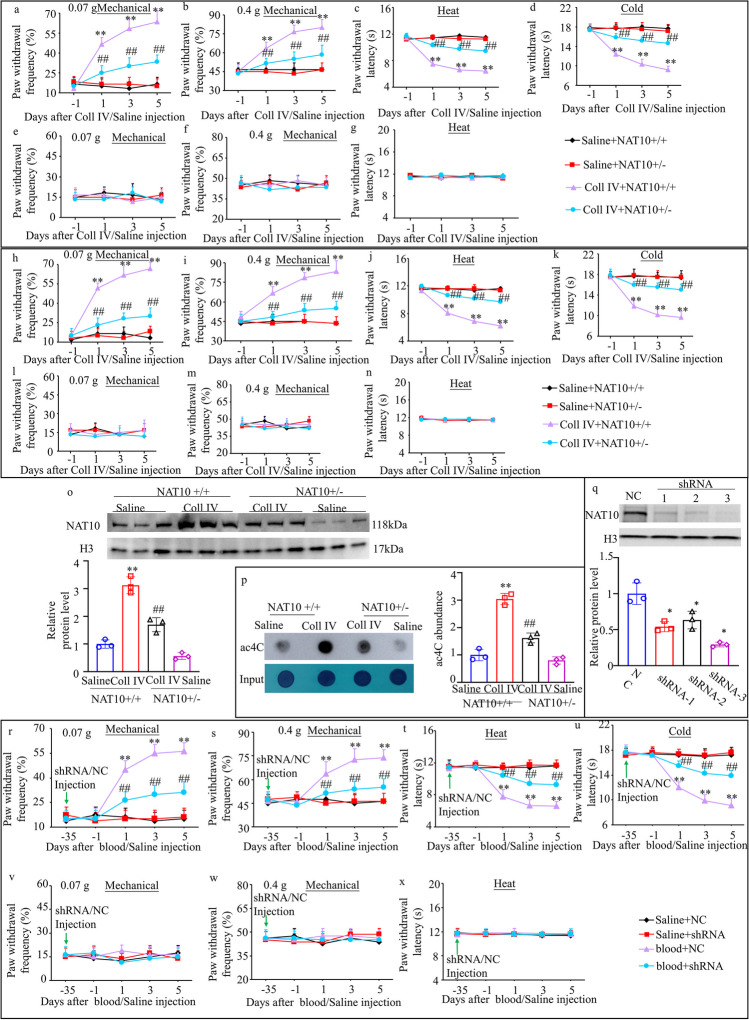
Effect of thalamic NAT10 knockdown in mice on Coll IV induced thalamic pain. Effect of thalamic NAT10 knockdown using transgenesis technology on Coll IV induced thalamic pain in male (**a**–**g**) and female (**h**–**n**) mice. Effects of thalamic NAT10 knockdown on mechanical pain tested by 0.07 g (**a**, **e**, **h**, and **l**) and 0.4 g (**b**, **f**, **i**, and **m**) von Frey filaments, heat (**c**, **g**, **j**, and **n**) and cold (**d** and **k**) pain on days 1 to 5 after thalamic microinjection of Coll IV or saline on the contralateral (**a**–**d** and **h**–**k**) and ipsilateral (**e**–**g** and **l**–**n**) sides. **P < 0.01 versus the corresponding baseline (day -1). ##*P* < 0.01 versus the Coll IV plus NAT10^+/+^ group at the corresponding days. Effect of thalamic NAT10 knockdown using transgenesis technology on the expression of NAT10 (**o**) and the level of ac4C (**p**) in the ipsilateral thalamus on day 5 after Coll IV or saline microinjection. **P < 0.01 versus the corresponding saline plus NAT10^+/+^ group. ##*P* < 0.01 versus the corresponding Coll IV plus NAT10^+/+^ group. **q** Evaluation of Nat10-shRNAs by western blot. *P < 0.01 versus the scrambled shRNA (NC). **r**–**x** Effect of thalamic NAT10 knockdown using shRNA 5 weeks before autologous blood or saline microinjection on mechanical pain tested by 0.07 g (**r** and **v**) and 0.4 g (**s** and **w**) von Frey filaments, heat (**t** and **x**) and cold (**u**) pain on the contralateral (**s**–**v**) and ipsilateral (**v**–**x**) sides on days 1, 3, and 5 after autologous blood/saline microinjection. **P < 0.01 versus the corresponding baseline (day -35). ##*P* < 0.01 versus the blood plus control scrambled shRNA (NC)-treated group at the corresponding days. **a**–**n** and **r**–**x** *n* = 8 mice/group. **o** and **p** *n* = 3 mice/group. **a**–**n** and **r**–**x** Two-way ANOVA with repeated measures followed by post hoc Tukey’s test. **o**–**p** Two-way ANOVA followed by post hoc Tukey’s test

To ensure that the microinjection of inhibitor and shRNA did not affect our results, we used heterozygous Nat10^+/−^ mice. Nat10 knockdown was found to significantly suppress the increase in the paw withdrawal frequency when exposed to 0.07 g and 0.4 g von Frey filaments and the decrease in paw withdrawal latency when exposed to heat and cold stimuli on days 1, 3, and 5 post-microinjection with Coll IV on the contralateral side among both male and female mice (Fig. 5a–d and h–k). Additionally, as expected, NAT10 knockdown showed no influence on the basal paw withdrawal frequency or latency on the ipsilateral side with Coll IV treatment (Fig. 5e–g and l–n). As expected, NAT10 knockdown did not affect locomotor function (Supplementary Table 4).

Then, we harvested brain tissues after all the behavioral tests for Nissl staining to determine whether NAT10 knockdown influenced the formation of Coll IV-induced TH lesions. NAT10 knockdown significantly increased the Nissl-stained cell count in the ipsilateral thalamus after Coll IV treatment (Supplementary Fig. 5a and b). As expected, there were no differences between the two groups in the Nissl-stained cell count in the contralateral thalamus (Supplementary Fig. 6a and b).

Brain samples were also obtained following the behavioral assessments to conduct a Western blotting analysis. In the ipsilateral thalamus, the NAT10^+/−^ plus Coll IV mice exhibited lower levels of NAT10 protein compared with the NAT10^+/+^ plus Coll IV mice (Fig. 5o). In line with these results, the ac4C level in the ipsilateral thalamus was lower in the NAT10^+/−^ plus Coll IV mice than in the NAT10^+/+^ plus Coll IV mice (Fig. 5p).

The protein levels of NAT10 decreased by shRNA3 was the greatest among the three sequences, and thus it was chosen for subsequent experiments (Fig. 5r). A similar behavioral response was observed after NAT10 knockdown via AAV9-shRNA microinjection into the thalamic VPL/VPM in a model of thalamic pain induced by autologous whole-blood microinjection (Fig. 5r-s and Supplementary Table 4).

### Thalamic NAT10 Overexpression Resulted in CPSP Symptoms

**Fig. 6:**
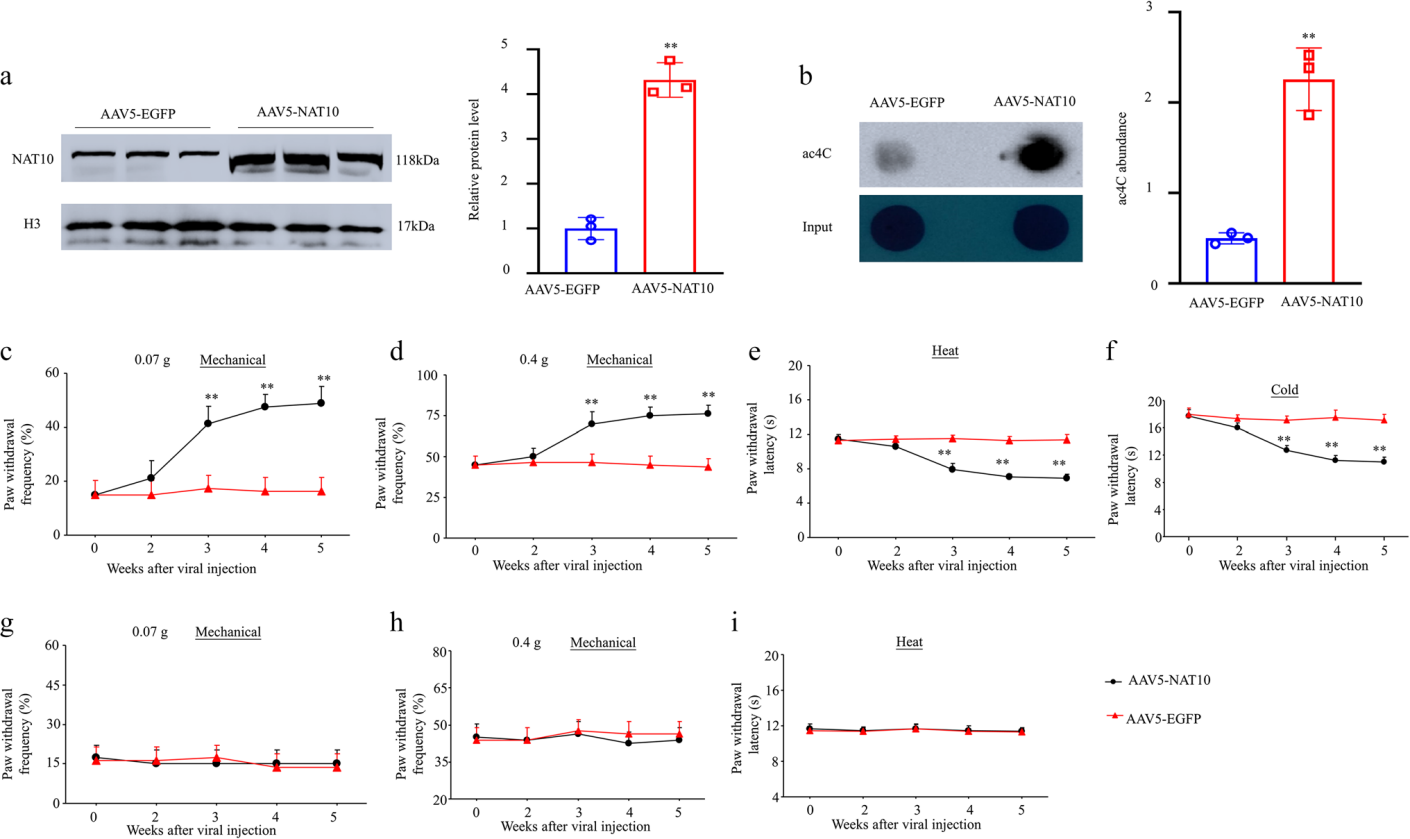
The impact of adeno-associated virus 5 (AAV5)-NAT10 microinjection into the unilateral thalamus to induce thalamic NAT10 overexpression on nociceptive thresholds in naïve mice. **a** and **b** The NAT10 protein (**a**) and ac4C modification (**b**) levels in the Ipsi thalamus 5 weeks post-viral microinjection. *n* = 3 biological repeats/group. **P < 0.01 versus the AAV5-EGFP group by 2-tailed unpaired Student’s *t* test. **c**–**i** Impact of the AAV5-NAT10 or AAV5-EGFP (as a control) microinjection into the VPL and VPM of the unilateral thalamus on paw withdrawal frequencies to 0.07 g (**c** and **g**) and 0.4 g (**d** and **h**) von Frey filaments and on the latencies of paw withdrawal in response to heat (**e** and **i**) and cold (**f**) stimuli on the contra (**c**–**f**) and Ipsi (**g**–**i**) sides at various weeks post-viral microinjection. *n* = 8 mice/group. Two-way ANOVA followed by post hoc Tukey’s test. **P < 0.01 versus the AAV5-EGFP group at the corresponding time points

The impact of NAT10 overexpression in the thalamus on basal nociceptive thresholds was subsequently investigated; NAT10 was overexpressed in naïve adult male mice by microinjecting the unilateral VPL and VPM of the thalamus with AAV5 expressing full-length NAT10 mRNA (AAV5-NAT10), with AAV5-EGFP serving as a control. Notably, a substantial increase in the amount of NAT10 protein (Fig. 6a) and the level of ac4C modification (Fig. 6b) in the injected thalamus were detected 5 weeks post-microinjection with AAV5-NAT10 relative to levels post-injection with AAV5-EGFP. Furthermore, when compared to microinjecting the mice with AAV5-EGFP, microinjecting them with AAV5-NAT10 resulted in a significant increase in the paw withdrawal frequency when exposed to 0.07 and 0.4 g von Frey filament stimuli (Fig. 6c and d) and a substantial reduction in the paw withdrawal latency in response to heat and cold stimuli on the contralateral side (Fig. 6e and f). Additionally, as predicted, microinjection of AAV5-NAT10 showed no impact on the basal paw withdrawal frequency or latency on the ipsilateral side during the observation period (Fig. 6g–i).

Administering Remodelin at 10 mg/kg (but not vehicle) through the tail vein once per day for 5 days beginning at 5 weeks post-viral microinjection resulted in a significant decrease in these types of nociceptive hypersensitivity on days 3 and 5 after Remodelin treatment (Supplementary Fig. 7). Systemic Remodelin administration had no remarkable impact on locomotor function, as anticipated (Supplementary Table 4). Taken together, thalamic NAT10 overexpression led to nociceptive hypersensitivity, which was reversed by Remodelin administration.

### Increased NAT10 Is Involved in TH-Induced Fn14 Upregulation and NF-κB Pathway Activation in the Thalamus

To identify the downstream target genes of NAT10 implicated in CPSP after TH, the NAT10-associated transcriptome was thoroughly characterized through the implementation of RNA immunoprecipitation (RIP-seq) on complexes containing NAT10. Notably, 656 transcripts exhibited a significantly altered abundance in animals with thalamic pain induced by hemorrhage, as determined by sequencing the RNAs associated with NAT10 (Supplementary Fig. 8a). Of these identified RNAs, protein-coding transcripts comprised 41.15% of the total, long intervening noncoding RNAs (lincRNAs) comprised 9.94%, antisense RNAs made up 5.11%, and other noncoding RNAs comprised 43.8% (Supplementary Fig. 8b). KEGG analysis revealed that these DEGs were related to the NF-κB, PI-3 K, and HIF-1 signalling pathways (Supplementary Fig. 8c).

**Fig. 7:**
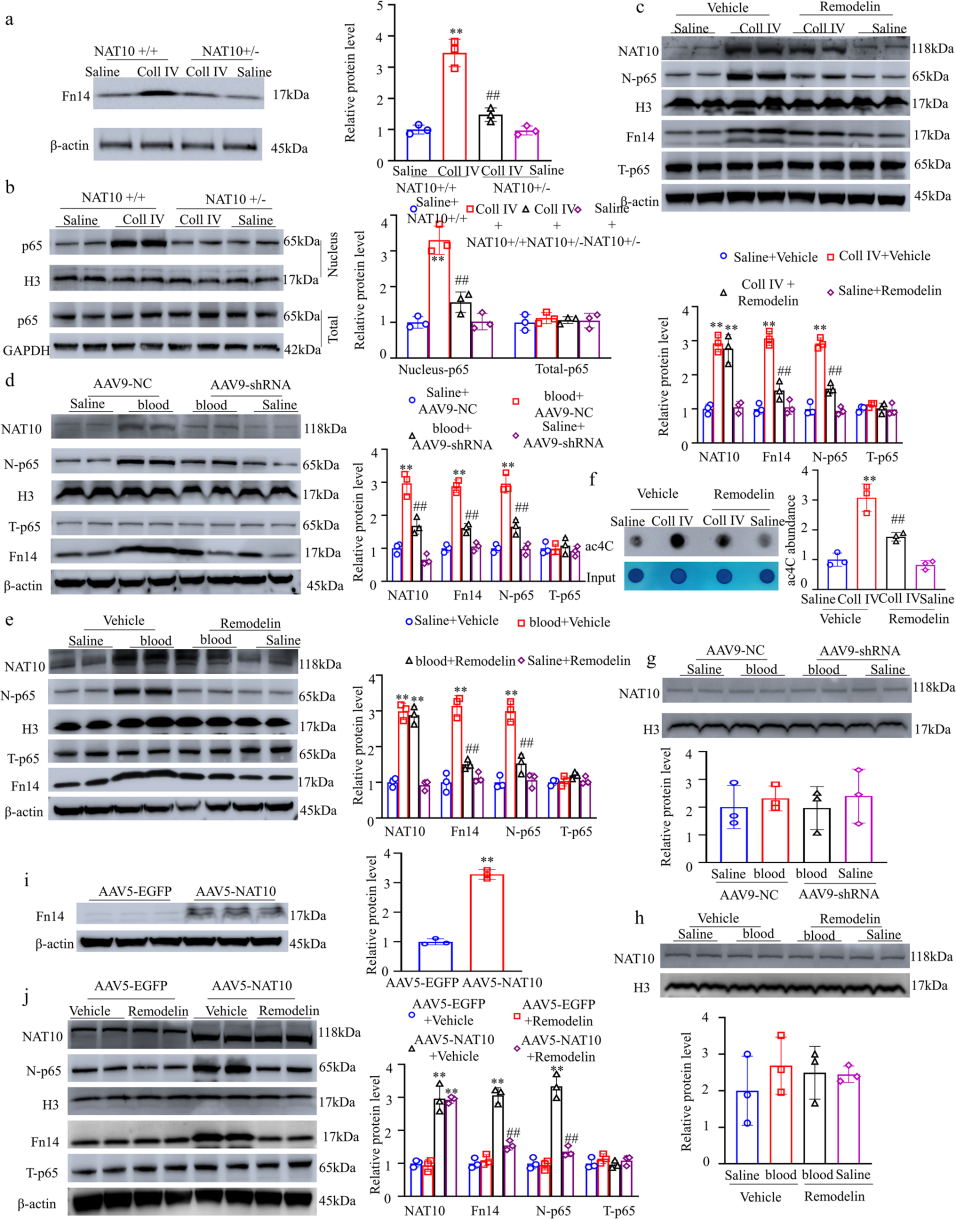
Increased NAT10 is involved in TH-induced Fn14/NF-κB pathway activation in the thalamus. Expression of Fn14 in the cytoplasmic fraction (Fn14) (**a**), total p65 in the total cellular fraction (T-p65) and p65 in the nuclear fraction (N-p65) (**b**) from the ipsilateral thalamus on day 5 after the Coll IV microinjection. **P < 0.01 versus the corresponding saline plus NAT10^+/+^ group. ##*P* < 0.01 versus the corresponding Coll IV plus NAT10^+/+^ group. Expression of NAT10 and p65 (N-p65) in the nuclear fraction; expression of Fn14; expression of T-p65 (**c**); and the level of ac4C (**f**) in the ipsilateral thalamus on day 5 after Coll IV microinjection with systemic preadministration of Remodelin (10 mg/kg). **P < 0.01 versus the corresponding saline plus vehicle-treated group. ##*P* < 0.01 versus the corresponding Coll IV plus vehicle-treated group. Expression of NAT10, N-p65, Fn14, and T-p65 from the ipsilateral thalamus (**d**) and NAT10 from the contralateral thalamus (**g**) on day 5 after the thalamic microinjection of autologous blood or saline. **P < 0.01 versus the corresponding saline plus AAV9-NC-treated group. ##*P* < 0.01 versus the corresponding blood plus AAV9-NC-treated group. Expression of NAT10, N-p65, Fn14, and T-p65 (**e**) from the ipsilateral thalamus and NAT10 from the contralateral thalamus (**h**) on day 5 after the autologous blood microinjection with systemic preadministration of Remodelin (10 mg/kg). **P < 0.01 versus the corresponding saline plus vehicle-treated group. ##*P* < 0.01 versus the corresponding blood plus vehicle-treated group. Expression of Fn14 in the cytoplasmic fraction from the ipsilateral thalamus on day 40 after the thalamic microinjection of AAV5-NAT10 or AAV5-EGFP (**i**). **P < 0.01 versus the AAV5-EGFP group. Expression of NAT10, N-p65, Fn14, and T-p65 (**j**) from the ipsilateral thalamus on day 40 after the thalamic microinjection of AAV5-NAT10 or AAV5-EGFP with administration of Remodelin (10 mg/kg). **P < 0.01 versus the corresponding AAV5-EGFP plus vehicle-treated group. ##*P* < 0.01 versus the corresponding AAV5-NAT10 plus vehicle-treated group. **a**–**j** Left or top: representative Western blots; right and bottom: statistical summary following densitometric analysis. **a**–**j** *n* = 3 mice/group. **a**–**h** and **j** Two-way ANOVA followed by post hoc Tukey’s test. **i** Two-tailed unpaired Student’s *t* test

Among these DEGs, we focused on *tnfrsf12a* (Supplementary Fig. 8d); the protein encoded by this gene, Fn14, can mediate the onset and progression of neuropathic pain and stroke by activating the NF-κB signalling pathway [26]. Based on the above sequencing analyses, we predicted that increased thalamic NAT10 may induce an increase in Fn14 levels. Indeed, in control mice, the Fn14 protein level was increased on day 5 post-microinjection with Coll IV relative to saline post-treatment (Fig. 7a). Furthermore, NAT10 knockout in the mice significantly blocked the upregulation of Fn14 protein mediated by the microinjection of Coll IV on day 5 post-treatment with Coll IV (Fig. 7a).

Moreover, in control mice, elevated levels of p65, a critical member of the NF-κB family, in the nuclear fraction on day 5 following Coll IV treatment, demonstrated that the NF-κB pathway was activated. However, NAT10 knockout significantly blocked the elevated concentration of p65 caused by the microinjection of Coll IV (Fig. 7b). Similar Fn14 and p65 expression patterns in the ipsilateral thalamus were observed after systemic Remodelin preadministration via tail vein infusion in the model of thalamic pain caused by Coll IV microinjection (Fig. 7c); similar Fn14 and p65 expression patterns in the ipsilateral thalamus were also observed after administration of shRNA targeting NAT10 or preadministration of Romodelin in a model of thalamic pain caused by autologous whole-blood microinjection (Fig. 7d and e). Compared with the Vehicle + Coll IV group, systemic preadministration of Remodelin significantly reduced the ac4C mRNA level in the model of thalamic pain caused by microinjecting the mice with Coll IV (Fig. 7f). In addition, neither thalamic microinjection of scrambled shRNA or NAT10 shRNA nor systemic preadministration of vehicle or Remodelin via tail vein injection was shown to alter the basal NAT10 expression in the contralateral thalamus (Fig. 7 g and h).

Consistent with these observations, mice microinjected with AAV5-NAT10 exhibited significantly elevated levels of the Fn14 and p65 proteins in response to thalamic NAT10 overexpression in the nuclear fraction in the injected thalamus as opposed to the levels in animals microinjected with AAV5-EGFP (Fig. 7i and j). Furthermore, this increase was abrogated after the administration of Remodelin via tail vein injection (Fig. 7j). Double immunohistochemical staining revealed that NAT10, Fn14, and p65 overlapped each other in the areas surrounding the core of hemorrhagic lesions in the thalamus on day 5 post-microinjection of Coll IV (Supplementary Fig. 9). In summary, these results demonstrated that NAT10 knockout inhibited the increase in p65 level induced by Coll IV microinjection, while NAT10 overexpression enhanced Fn14 and p65 protein levels in the thalamic pain model.

## Discussion

CPSP is a chronic form of central neuropathic pain that occurs after a stroke, characterized by intractable pain and commonly seen as a consequence of stroke. The origin and development of CPSP are not fully understood, and finding effective prevention and treatment methods for CPSP remains a key focus in clinical practice. Using a mouse model of TH-induced CPSP, we found that TH increased ac4C mRNA modification and identified NAT10 as a mediator of central pain after TH via the upregulation of Fn14 through the NF-κB pathway.

RNA modification is currently a hot topic in epigenetic research and performs an essential function in physiological and pathological processes through posttranscriptional regulation [8, 11]. The modification of mRNA with ac4C is a newly discovered type of mRNA modification. Studies have shown that ac4C modification can occur on multiple transcripts and can improve transcript stability and translation efficiency [10]. According to these findings, modification of ac4C mRNA could exert a significant impact on pathological.

In this study, the acRIP-seq analysis revealed significant changes in ac4C mRNA levels following TH. Specifically, we noted an elevation in the ac4C mRNA levels of 922 genes, while the ac4C mRNA levels of 132 genes were downregulated compared to the control group. These findings are consistent with our subsequent ac4C dot blot experiments, which further confirmed the sustained increase in ac4C levels after TH. Importantly, the above results suggest that the modification of mRNA with ac4C may play an important role in CPSP after TH.

Neuronal function plays a pivotal role in CPSP after TH. There is good evidence that thalamic pain occurs due to the increased glutamate release from synapses, overstimulation of glutamate postsynaptic excitatory receptors, and eventual influx of large amounts of Ca^2+^ through these receptors into thalamic neurons after hemorrhage [27]. By pharmacologically inhibiting NAT10 function via the NAT10 inhibitor Remodelin, we found that increased NAT10 expression is involved in the occurrence and maintenance of hyperalgesia after TH and that pharmacological inhibition of NAT10 function can significantly and dosage-dependently improve hyperalgesia. To verify the role of NAT10 in CPSP after TH via multiple methods, we found that the use of AAV9-shRNA could rescue hyperalgesia after TH caused by autologous whole-blood microinjection. Additionally, we found that in the NAT10^±^ mouse CPSP model, the development of CPSP in female and male mice was significantly alleviated due to the downregulation of NAT10. Specific pathways in spinal microglia or dorsal root ganglion macrophages are involved in sexual dimorphism in neuropathic pain [28, 29]. Sexual dimorphism appears to be restricted to microglia or macrophages, as the suppression of pain-associated signalling in neurons and astrocytes produces comparable analgesic effects in both sexes [30, 31]. Given that NAT10 is primarily expressed in thalamic neurons, these findings explain the male and female mice in this study exhibited consistent pain symptoms.

We conducted NAT10-RIP experiments to search for the downstream molecules on which NAT10 acts. Among the many target molecules, Tnfrsf12a attracted our attention. Tnfrsf12a encodes Fn14, which is expressed in endothelial cells, astrocytes, microglia, and neurons in the central nervous system. Fn14 is critically involved in the onset of neurological disorders, such as neuropsychiatric diseases, cerebral ischemia, and multiple sclerosis [26, 32, 33]. For example, during cerebral ischemia, Fn14 expression is upregulated, and early inhibition of Fn14 expression has significant neuroprotective effects [33]. Recent research has indicated that the stimulation of the NF-κB signalling pathway via the upregulation of Fn14 expression might have a significant impact on the occurrence and maintenance of neuropathic pain [34]. This study illustrated that Fn14 protein expression increased after TH, and the upregulation of Fn14 expression could be reversed after pharmacological inhibition of NAT10 function or knockdown of NAT10 by AAV9-shRNA and in NAT10^+/−^ mice with downregulated NAT10 expression. Similarly, Fn14 protein expression was upregulated after NAT10 was overexpressed by using AAV5-NAT10. The change in Fn14 protein expression after TH was consistent with the change in NAT10 expression.

In 2018, Cell reported for the first time that mRNA acetylation modification can promote translation efficiency by stable mRNA [10]. In addition, our results showed that the upregulation of NAT10 increases Tnfrsf12a ac4C mRNA levels, which may increase the stability and translation efficiency of Tnfrsf12a mRNA, resulting in increased expression of its translation product, the Fn14 protein. The above research results suggest that the upregulation of NAT10 after TH may mediate the occurrence of CPSP after TH by increasing the level of Tnfrsf12a mRNA modification with ac4C, which in turn leads to an increase in Fn14 protein expression. However, we need to focus on a primary limitation, namely, that other potential mechanisms involving NAT10 in CPSP induced after TH cannot be excluded. Through an ac-RIP assay, we revealed that 922 genes had increased ac4C mRNA levels and that 132 genes had downregulated ac4C mRNA levels after TH. Previous articles indicated that it is necessary to further investigate the role of Fn14 in CPSP. Despite this, there are several mechanisms via which NAT10 might exacerbate thalamic pain caused by hemorrhage., which deserve further study, like protein–protein interaction networks.

NF-κB is a crucial nuclear transcriptional factor in the development of peripheral neuropathic pain, as it regulates multiple genes that encode nociceptive mediators and inflammatory cytokines [35, 36]. Intrathecal pyrrolidine dithiocarbamate, an NF-κB inhibitor, reduces neuropathic pain and neuroinflammation [37–39]. Our previous studies showed that the NF-κB signalling pathway is a significant factor in CPSP following TH [17, 19]. NF-κB signalling pathway activation by upregulated Fn14 may contribute significantly to the occurrence and maintenance of neuropathic pain [34]. The study illustrated that blocking the upregulation of NAT10 expression after TH significantly decreased the translocation of the NF-κB subunit p65 into the nucleus after TH while downregulating Fn14 expression. Since the NAT10, Fn14, and p65 proteins were found by immunofluorescence staining to colocalize, NAT10 is likely needed to upregulate Fn14 and thereby activate NF-κB signalling during the pathophysiological process of CPSP induced by TH. It is undetermined whether increased NAT10 and Fn14 after TH stimulate other cellular signals. However, Fn14 is at least partially responsible for thalamic pain by triggering the NF-κB pathway in thalamic neurons.

Another key limitation is the correlation among NAT10, Fn14, and NF-κB lacks a more rigorous demonstration. Specific shRNA or Enavatuzumab can be used to inhibit Fn14 receptor [40, 41] to strengthen the role of Fn14 in the process by which ac4C modification induces CPSP. In addition, when designing the NAT10 primer, we found it impossible to differentiate its encoding mRNA from the noncoding RNA, whose sequence overlaps with all mRNA sequences. However, this limitation does not affect the conclusion because data from RNA-seq and Western blot are most critical in supporting the increase in NAT10 under TH.

Our research in mice has identified NAT10 as a key modulator of pain pathways, a discovery that could have significant translational potential. The mechanisms by which NAT10 influences pain signalling offer a novel avenue for therapeutic intervention, possibly leading to the development of targeted treatments that could alleviate the debilitating symptoms of CPSP in humans. However, the translation of these findings from mice to humans is not without its challenges. An anticipated challenge is the specificity of any intervention targeting NAT10. Given the potential for pleiotropic effects, it is crucial to develop treatments that can modulate NAT10’s activity without disrupting other essential biological processes. This necessitates a thorough understanding of the molecular context in which NAT10 operates and the development of highly specific drugs or interventions.

In conclusion, our results reveal that NAT10 expression and ac4C levels increase after TH. NAT10 regulates Fn14 protein expression through ac4C modification and performs a critical function in CPSP after TH. To our knowledge, this study provides the first evidence that NAT10-regulated RNA ac4C modification is closely related to CPSP after TH and will undoubtedly provide new ideas and new ways to identify effective methods for treating CPSP that target NAT10.

## Supplementary Information

Below is the link to the electronic supplementary material.

Supplementary Fig. 1 Thalamic haemorrhage results in pain hypersensitivity. The microinjection of collagenase IV (Coll IV) into the ventral posterior medial nuclei and ventral posterior lateral nuclei resulted in an increased paw withdrawal frequency in response to 0.07 g (a) and 0.4 g (b) von Frey filaments and a decreased paw withdrawal latency in response to thermal (c) and cold (d) stimuli on the contralateral side. There were no observed alterations in paw withdrawal frequency (e and f) or latency (g) on the ipsilateral side. n = 8 mice per group. Two-way ANOVA with repeated measures followed by post hoc Tukey’s test. **P < 0.01 versus the saline-treated group at the corresponding time points (PNG 492 kb)

High resolution image (TIF 4034 kb)

Supplementary Fig. 2 Effect of the Remodelin (10 mg/kg) on Coll IV induced thalamic lesions. (a) Representative coronal brain sections stained with Nissl from the different treatment groups on day 5 after the thalamic microinjection of Coll IV or saline. Top: brain sections, including the ventral posterior lateral nucleus (VPL) and ventral posterior medial nucleus (VPM); scale bar: 100 μm. Bottom: magnification of the corresponding photographs in the top row; scale bar: 50 μm. (b) Number of Nissl-stained cells in the VPL and VPM of thalami from the different indicated treatment groups. n = 3 biological repeats/group. Two-way ANOVA followed by post hoc Tukey’s test. **P < 0.01 versus the saline plus vehicle–treated group. ##P < 0.01 versus the Coll IV plus vehicle–treated group (PNG 1056 kb)

High resolution image (TIF 19642 kb)

Supplementary Fig. 3 Effect of Remodelin (10 mg/kg) on Nissl-stained cells in the contralateral thalamus. (a) Representative coronal brain sections stained with Nissl from the different treatment groups on day 5 after thalamic Coll IV or saline microinjection. Top: brain sections, including the VPL and VPM; scale bar: 100 μm. Bottom: magnification of the corresponding photographs in the top row; scale bar: 50 μm. (b) Number of Nissl-stained cells in the VPL and VPM of thalami from the different indicated treatment groups. n = 3 biological repeats/group. Two-way ANOVA followed by post hoc Tukey’s test (PNG 1175 kb)

High resolution image (TIF 21482 kb)

Supplementary Fig. 4 Effect of systemic tail vein administration of Remodelin on autologous blood microinjection-induced thalamic pain genesis. Remodelin or vehicle was given 30 min before autologous blood or saline microinjection and once daily thereafter. Effects of systemic administration of Remodelin (10 mg/kg) or vehicle on paw withdrawal frequencies to 0.07 g (a and e) and 0.4 g (b and f) von Frey filaments and on paw withdrawal latencies to heat (c and g) and cold (d) stimuli on days 1 to 5 after thalamic microinjection of autologous blood or saline on the contralateral (a-d) and ipsilateral (e-g) sides. n = 8 mice/group. Two-way ANOVA with repeated measures followed by post hoc Tukey’s test. **P < 0.01 versus the corresponding baseline (day -1). ##P < 0.01 versus the blood plus vehicle-treated group at the corresponding days (PNG 206 kb)

High resolution image (TIF 4231 kb)

Supplementary Fig. 5 Effect of thalamic NAT10 knockdown using transgenesis technology on Coll IV induced thalamic lesions. (a) Representative coronal brain sections stained with Nissl from the different treatment groups on day 5 after thalamic microinjection of Coll IV or saline. Top: brain sections, including the VPL and VPM; scale bar: 100 μm. Bottom: magnification of the corresponding photographs in the top row; scale bar: 50 μm. (b) Number of Nissl-stained cells in the VPL and VPM of thalami from the different indicated treatment groups. n = 3 biological repeats/group. Two-way ANOVA followed by post hoc Tukey’s test. **P < 0.01 versus the saline plus NAT10^+/+^ group. ##P < 0.01 versus the Coll IV plus NAT10^+/+^ group (PNG 974 kb)

High resolution image (TIF 18023 kb)

Supplementary Fig. 6 Effect of thalamic NAT10 knockdown using transgenesis technology on Nissl-stained cells in the contralateral thalamus. (a) Representative coronal brain sections stained with Nissl from the different treatment groups on day 5 after thalamic Coll IV or saline microinjection. Top: brain sections, including the VPL and VPM; scale bar: 100 μm. Bottom: magnification of the corresponding photographs in the top row; scale bar: 50 μm. (b) Number of Nissl-stained cells in the VPL and VPM of thalami from the different indicated treatment groups. n = 3 biological repeats/group. Two-way ANOVA followed by post hoc Tukey’s test (PNG 975 kb)

High resolution image (TIF 18220 kb)

Supplementary Fig. 7 Effect of Remodelin on nociceptive sensitivity in mice with central post-stroke pain. Effect of the systemic tail vein administration of Remodelin (10 mg/kg) once daily for 5 days beginning 35 days after viral microinjection on paw withdrawal frequencies in response to 0.07 g (a) and 0.4 g (b) von Frey filaments and paw withdrawal latencies in response to heat (c) and cold (d) stimuli on the contralateral side at the indicated time points in naïve mice. n = 8 mice/group. Two-way ANOVA with repeated measures followed by post hoc Tukey’s test. **P < 0.01 versus the corresponding baseline (day -35). ##P < 0.01 versus the AAV5-NAT10 plus vehicle-treated group at the corresponding days (PNG 179 kb)

High resolution image (TIF 4101 kb)

Supplementary Fig. 8 Identification of NAT10-associated sequences by acetylated RNA-binding protein immunoprecipitation sequencing analysis in mice with thalamic haemorrhage. (a) MA plot of the NAT10. (b) Classification of the NAT10-associated RNAs. (c) Heatmap of differentially expressed NAT10 target genes. (d) Functional enrichment analysis of NAT10 target genes. The bar plot displays Gene Ontology terms (BP, biological process; CC, cellular component; MF, molecular function) and Kyoto Encyclopedia of Genes and Genomes pathways enriched in the NAT10 target genes (PNG 318 kb)

High resolution image (TIF 5913 kb)

Supplementary Fig. 9 Coexpression of NAT10 with Fn14 or p65 and coexpression of p65 with Fn14. Double immunohistochemical staining revealed that NAT10 immunoreactivity overlapped with Fn14 and p65 immunoreactivity in the regions adjacent to the core of haemorrhagic lesions in the thalamus on day 5 after Coll IV microinjection. p65 immunoreactivity overlapped with Fn14 immunoreactivity in this region. Representative images from 3 biological repeats (n = 3 mice). Scale bar: 50 μm (PNG 477 kb)

High resolution image (TIF 10347 kb)

Supplementary file 10 (PNG 501 kb)

High resolution image (TIF 13362 kb)

Supplementary Tables 1-4 (DOC 62 kb)

## Data Availability

No datasets were generated or analysed during the current study.
