# Supplementary figures and images for: The Cytidine N-Acetyltransferase NAT10 Promotes Thalamus Hemorrhage-Induced Central Poststroke Pain by Stabilizing Fn14 Expression in Thalamic Neurons
Source: Mol Neurobiol. 2024 Sep 13;62(3):3276–92. doi: 10.1007/s12035-024-04454-4 (PMC11790786; doi:10.1007/s12035-024-04454-4)

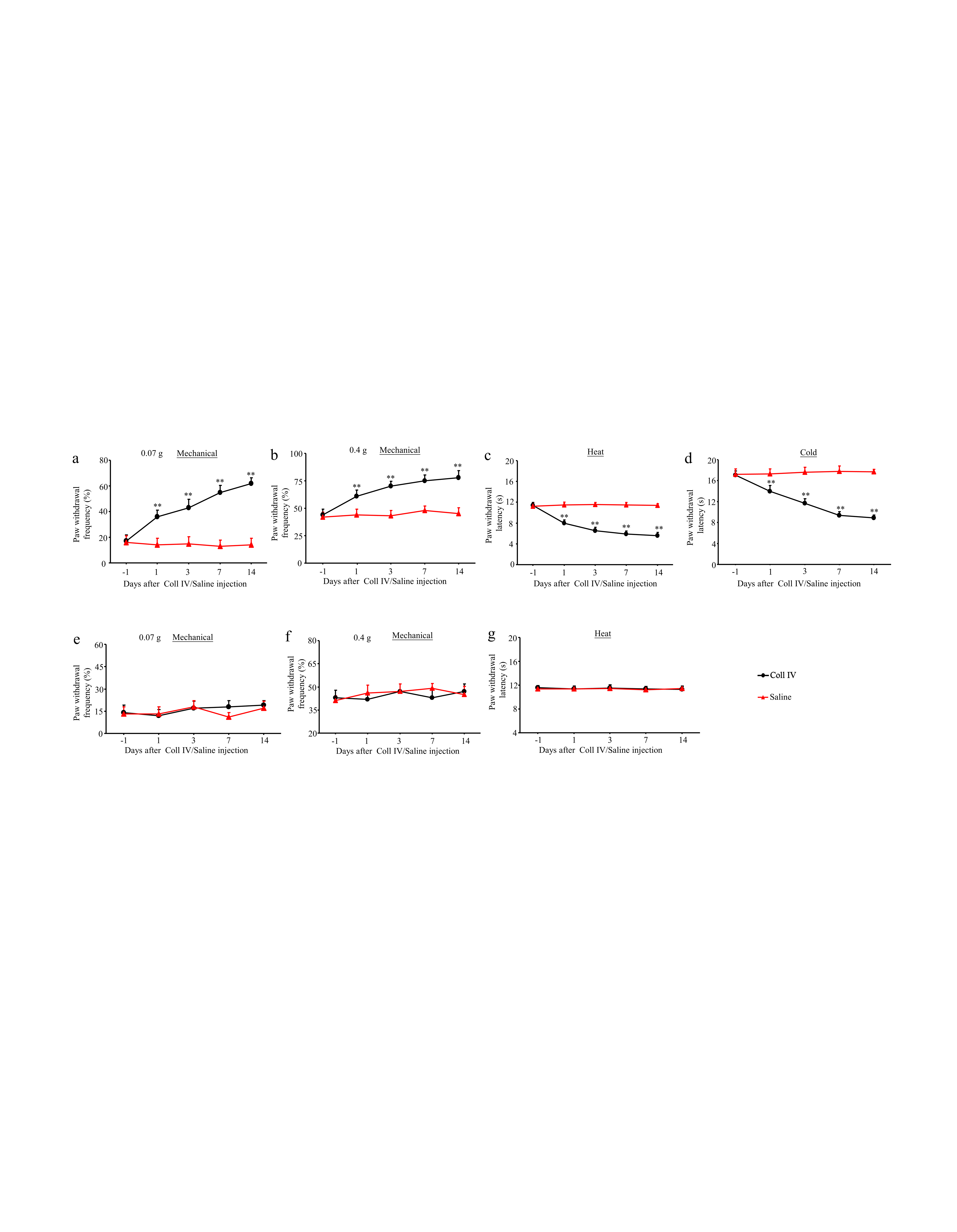

Supplement: Supplementary file 1 — Thalamic haemorrhage results in pain hypersensitivity. The microinjection of collagenase IV (Coll IV) into the ventral posterior medial nuclei and ventral posterior lateral nuclei resulted in an increased paw withdrawal frequency in response to 0.07 g (a) and 0.4 g (b) von Frey filaments and a decreased paw withdrawal latency in response to thermal (c) and cold (d) stimuli on the contralateral side. There were no observed alterations in paw withdrawal frequency (e and f) or latency (g) on the ipsilateral side. n = 8 mice per group. Two-way ANOVA with repeated measures followed by post hoc Tukey’s test. **P < 0.01 versus the saline-treated group at the corresponding time points (PNG 492 kb) [file 12035_2024_4454_Fig8_ESM.png]

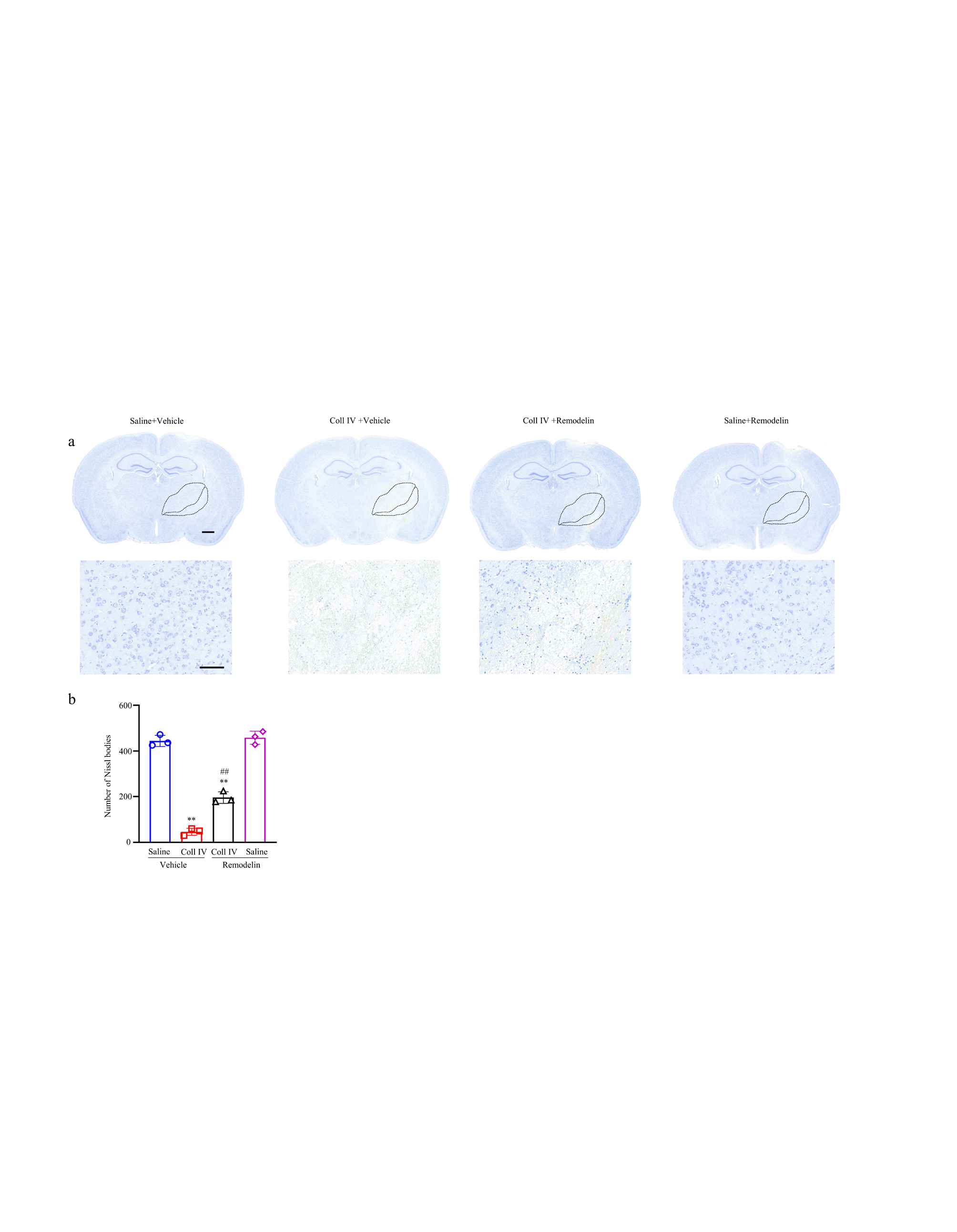

Supplement: Supplementary file 3 — Effect of the Remodelin (10 mg/kg) on Coll IV induced thalamic lesions. (a) Representative coronal brain sections stained with Nissl from the different treatment groups on day 5 after the thalamic microinjection of Coll IV or saline. Top: brain sections, including the ventral posterior lateral nucleus (VPL) and ventral posterior medial nucleus (VPM); scale bar: 100 μm. Bottom: magnification of the corresponding photographs in the top row; scale bar: 50 μm. (b) Number of Nissl-stained cells in the VPL and VPM of thalami from the different indicated treatment groups. n = 3 biological repeats/group. Two-way ANOVA followed by post hoc Tukey’s test. **P < 0.01 versus the saline plus vehicle–treated group. ##P < 0.01 versus the Coll IV plus vehicle–treated group (PNG 1056 kb) [file 12035_2024_4454_Fig9_ESM.png]

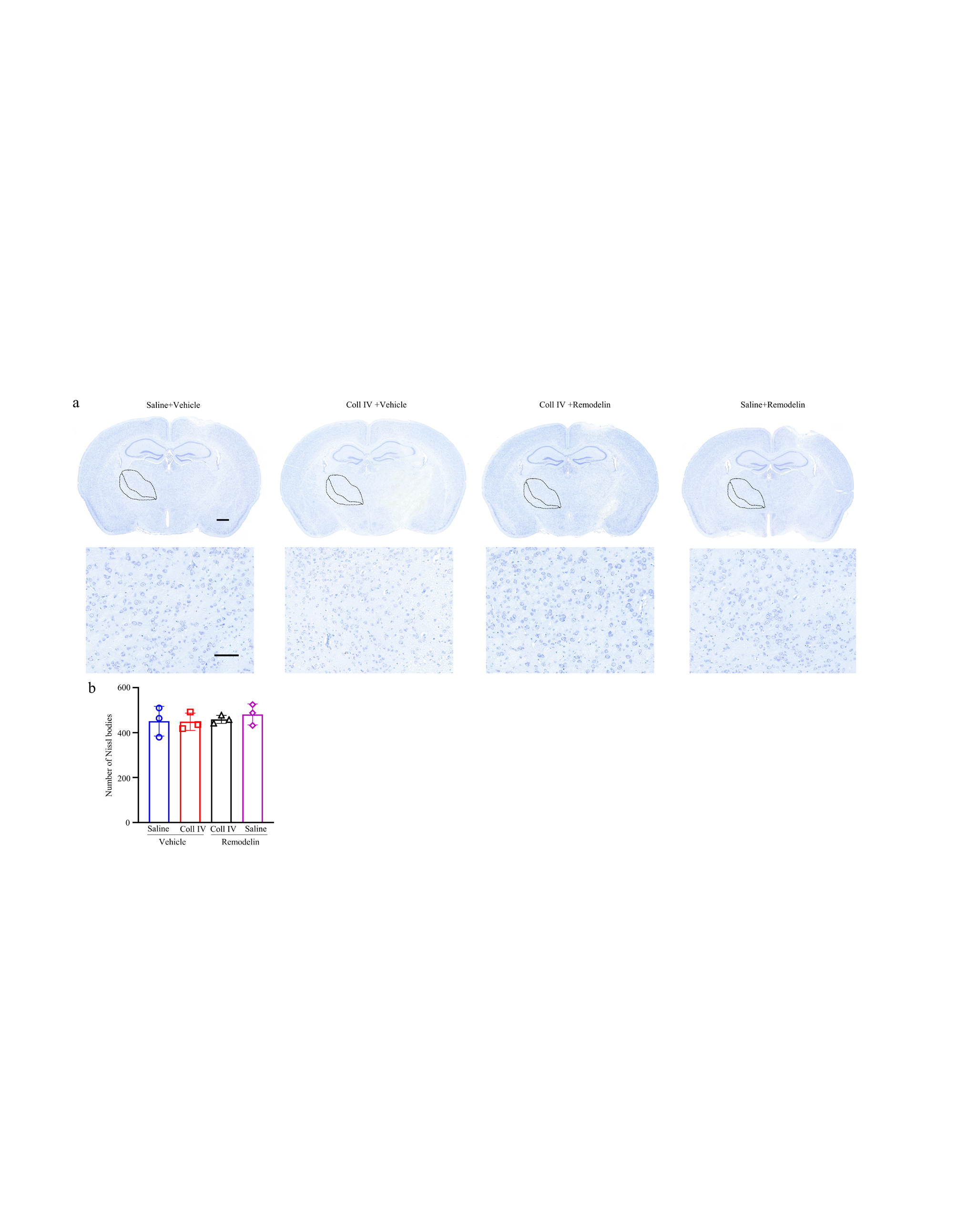

Supplement: Supplementary file 5 — Effect of Remodelin (10 mg/kg) on Nissl-stained cells in the contralateral thalamus. (a) Representative coronal brain sections stained with Nissl from the different treatment groups on day 5 after thalamic Coll IV or saline microinjection. Top: brain sections, including the VPL and VPM; scale bar: 100 μm. Bottom: magnification of the corresponding photographs in the top row; scale bar: 50 μm. (b) Number of Nissl-stained cells in the VPL and VPM of thalami from the different indicated treatment groups. n = 3 biological repeats/group. Two-way ANOVA followed by post hoc Tukey’s test (PNG 1175 kb) [file 12035_2024_4454_Fig10_ESM.png]

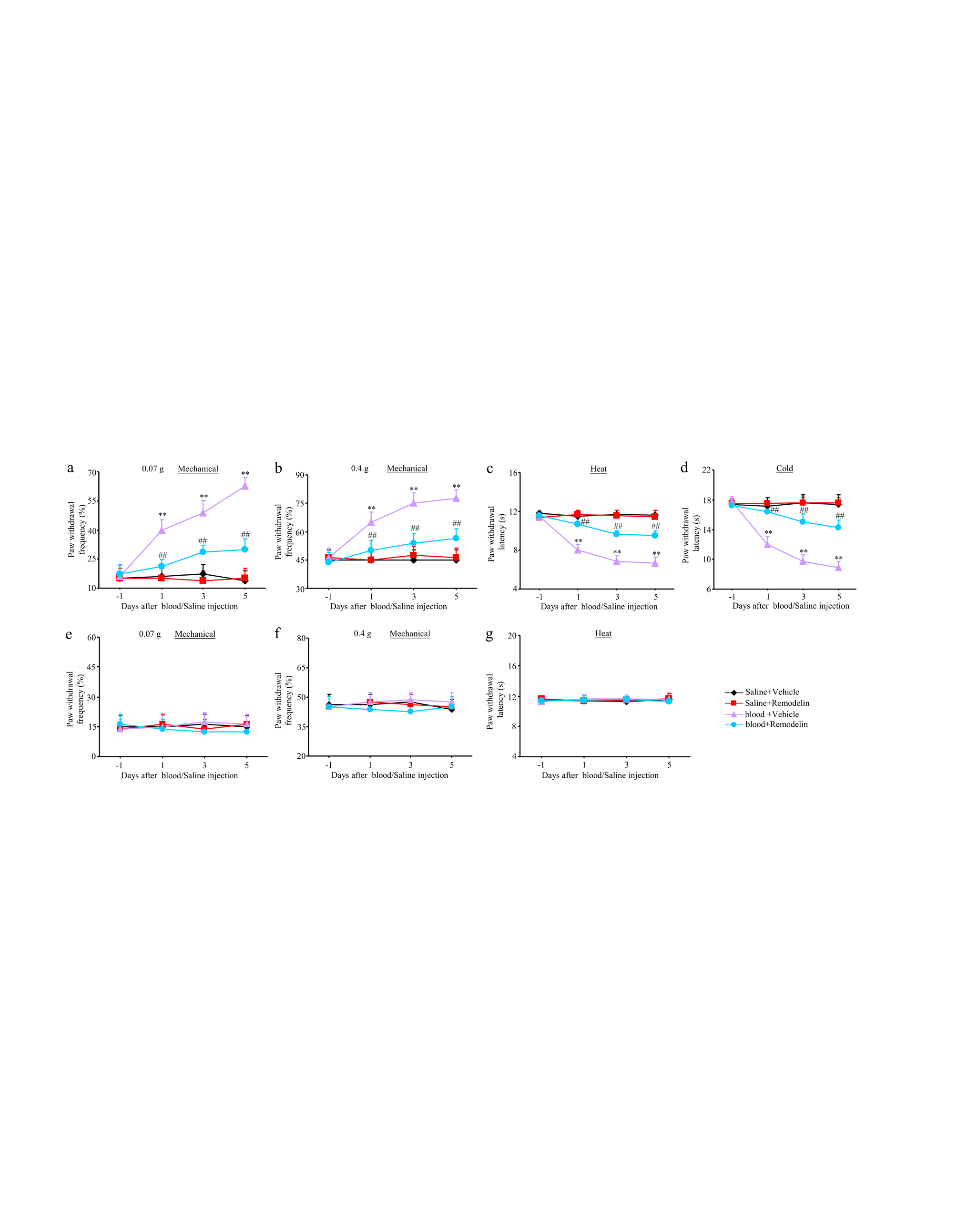

Supplement: Supplementary file 7 — Effect of systemic tail vein administration of Remodelin on autologous blood microinjection-induced thalamic pain genesis. Remodelin or vehicle was given 30 min before autologous blood or saline microinjection and once daily thereafter. Effects of systemic administration of Remodelin (10 mg/kg) or vehicle on paw withdrawal frequencies to 0.07 g (a and e) and 0.4 g (b and f) von Frey filaments and on paw withdrawal latencies to heat (c and g) and cold (d) stimuli on days 1 to 5 after thalamic microinjection of autologous blood or saline on the contralateral (a-d) and ipsilateral (e-g) sides. n = 8 mice/group. Two-way ANOVA with repeated measures followed by post hoc Tukey’s test. **P < 0.01 versus the corresponding baseline (day -1). ##P < 0.01 versus the blood plus vehicle-treated group at the corresponding days (PNG 206 kb) [file 12035_2024_4454_Fig11_ESM.png]

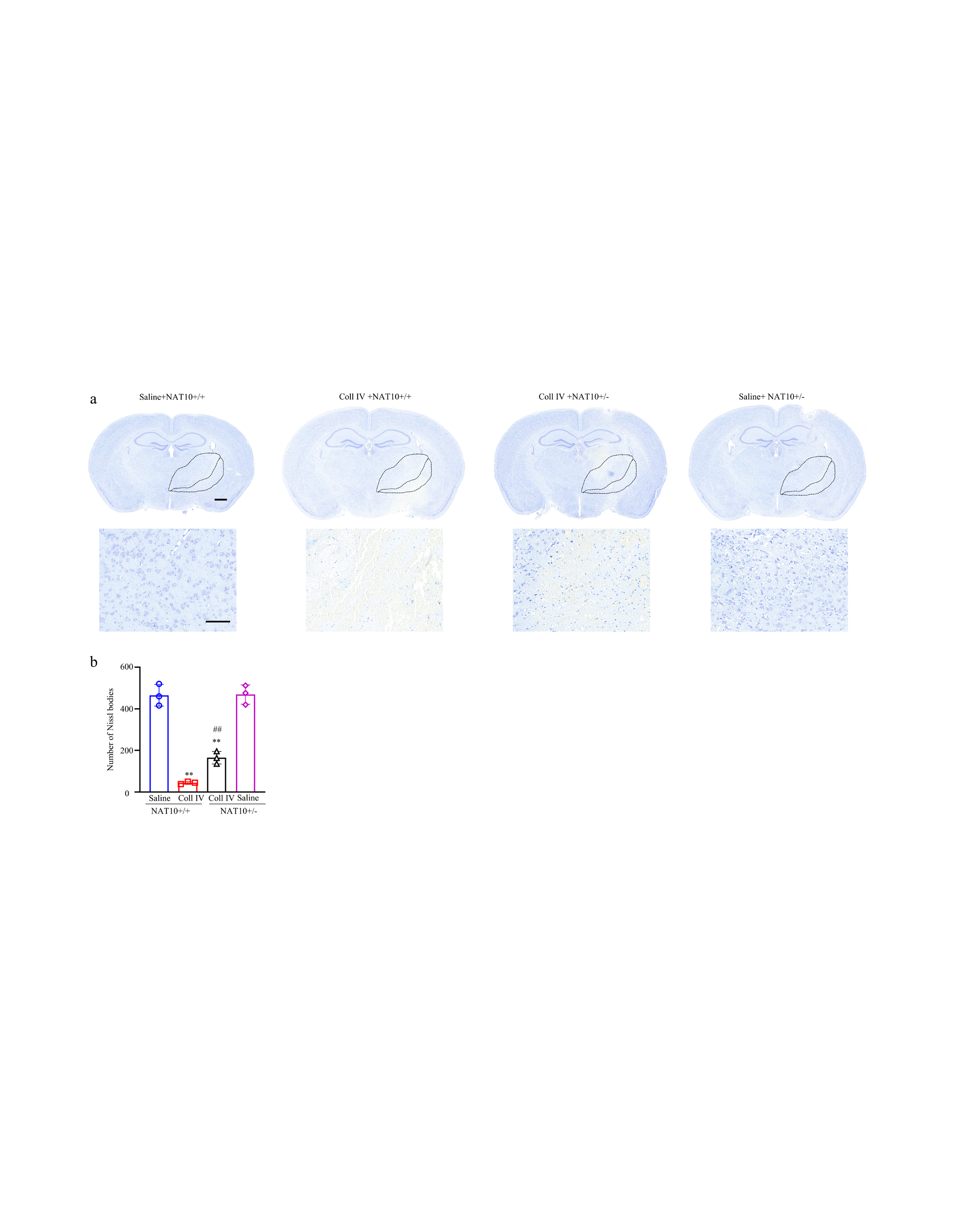

Supplement: Supplementary file 9 — Effect of thalamic NAT10 knockdown using transgenesis technology on Coll IV induced thalamic lesions. (a) Representative coronal brain sections stained with Nissl from the different treatment groups on day 5 after thalamic microinjection of Coll IV or saline. Top: brain sections, including the VPL and VPM; scale bar: 100 μm. Bottom: magnification of the corresponding photographs in the top row; scale bar: 50 μm. (b) Number of Nissl-stained cells in the VPL and VPM of thalami from the different indicated treatment groups. n = 3 biological repeats/group. Two-way ANOVA followed by post hoc Tukey’s test. **P < 0.01 versus the saline plus NAT10+/+ group. ##P < 0.01 versus the Coll IV plus NAT10+/+ group (PNG 974 kb) [file 12035_2024_4454_Fig12_ESM.png]

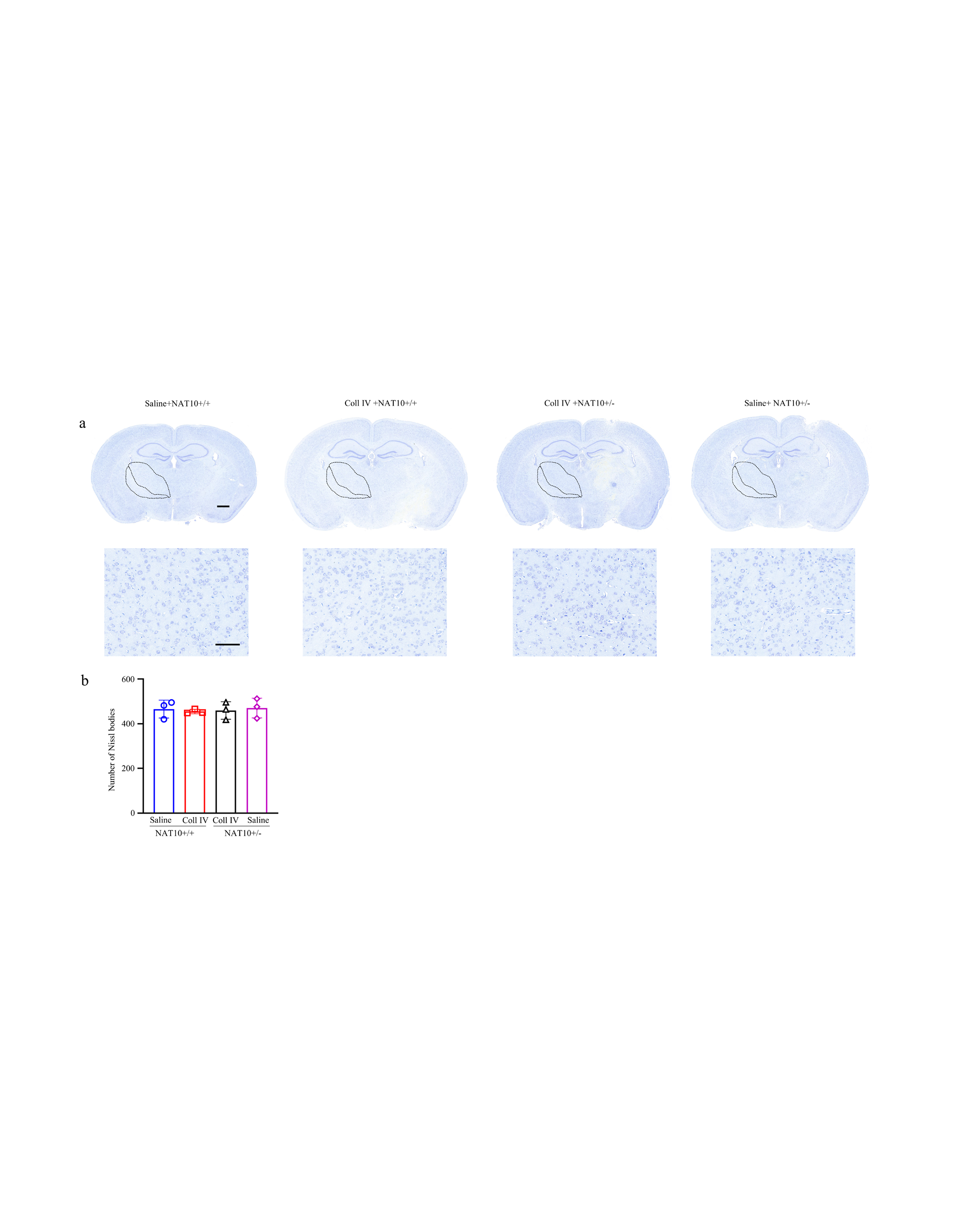

Supplement: Supplementary file 11 — Effect of thalamic NAT10 knockdown using transgenesis technology on Nissl-stained cells in the contralateral thalamus. (a) Representative coronal brain sections stained with Nissl from the different treatment groups on day 5 after thalamic Coll IV or saline microinjection. Top: brain sections, including the VPL and VPM; scale bar: 100 μm. Bottom: magnification of the corresponding photographs in the top row; scale bar: 50 μm. (b) Number of Nissl-stained cells in the VPL and VPM of thalami from the different indicated treatment groups. n = 3 biological repeats/group. Two-way ANOVA followed by post hoc Tukey’s test (PNG 975 kb) [file 12035_2024_4454_Fig13_ESM.png]

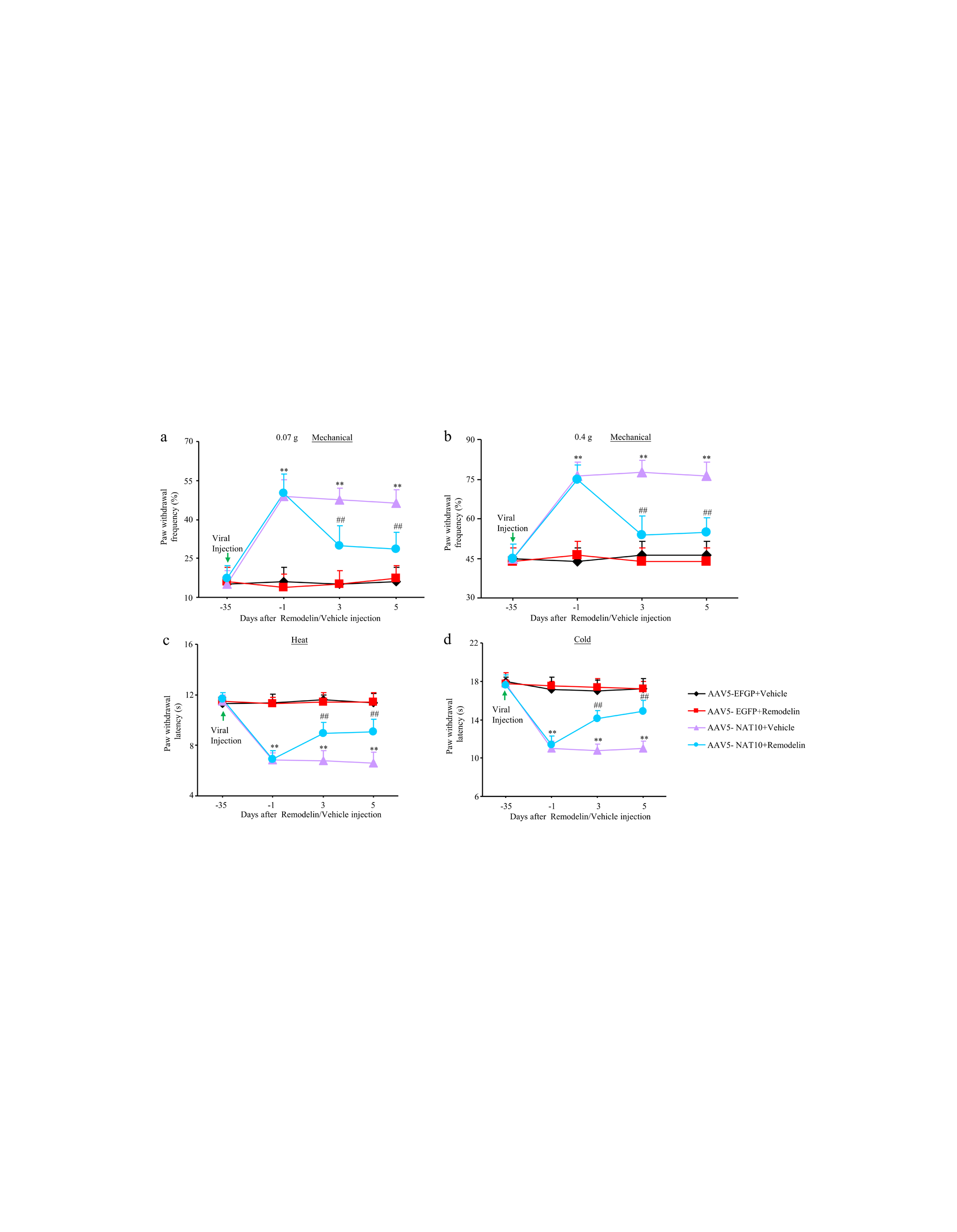

Supplement: Supplementary file 13 — Effect of Remodelin on nociceptive sensitivity in mice with central post-stroke pain. Effect of the systemic tail vein administration of Remodelin (10 mg/kg) once daily for 5 days beginning 35 days after viral microinjection on paw withdrawal frequencies in response to 0.07 g (a) and 0.4 g (b) von Frey filaments and paw withdrawal latencies in response to heat (c) and cold (d) stimuli on the contralateral side at the indicated time points in naïve mice. n = 8 mice/group. Two-way ANOVA with repeated measures followed by post hoc Tukey’s test. **P < 0.01 versus the corresponding baseline (day -35). ##P < 0.01 versus the AAV5-NAT10 plus vehicle-treated group at the corresponding days (PNG 179 kb) [file 12035_2024_4454_Fig14_ESM.png]

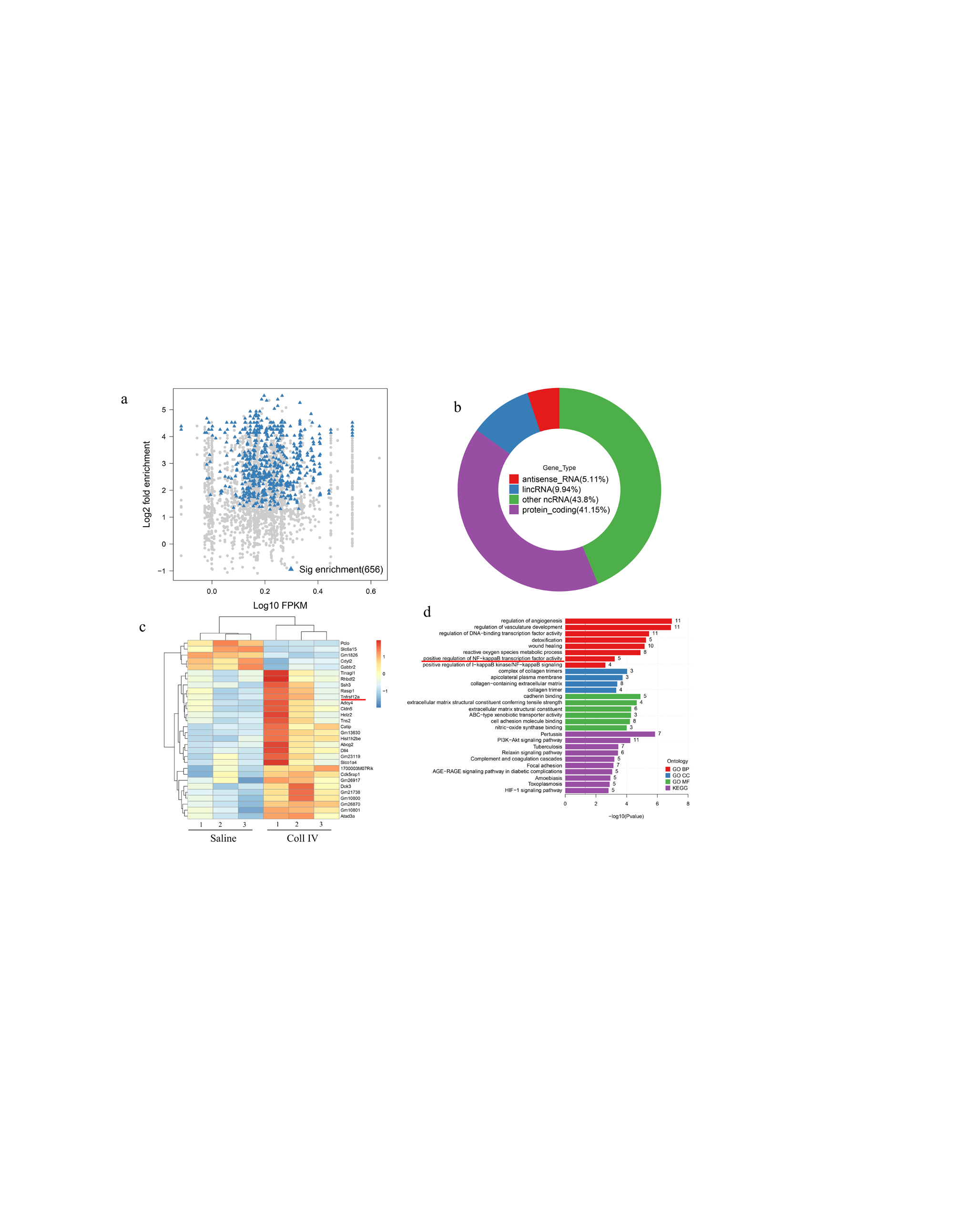

Supplement: Supplementary file 15 — Identification of NAT10-associated sequences by acetylated RNA-binding protein immunoprecipitation sequencing analysis in mice with thalamic haemorrhage. (a) MA plot of the NAT10. (b) Classification of the NAT10-associated RNAs. (c) Heatmap of differentially expressed NAT10 target genes. (d) Functional enrichment analysis of NAT10 target genes. The bar plot displays Gene Ontology terms (BP, biological process; CC, cellular component; MF, molecular function) and Kyoto Encyclopedia of Genes and Genomes pathways enriched in the NAT10 target genes (PNG 318 kb) [file 12035_2024_4454_Fig15_ESM.png]

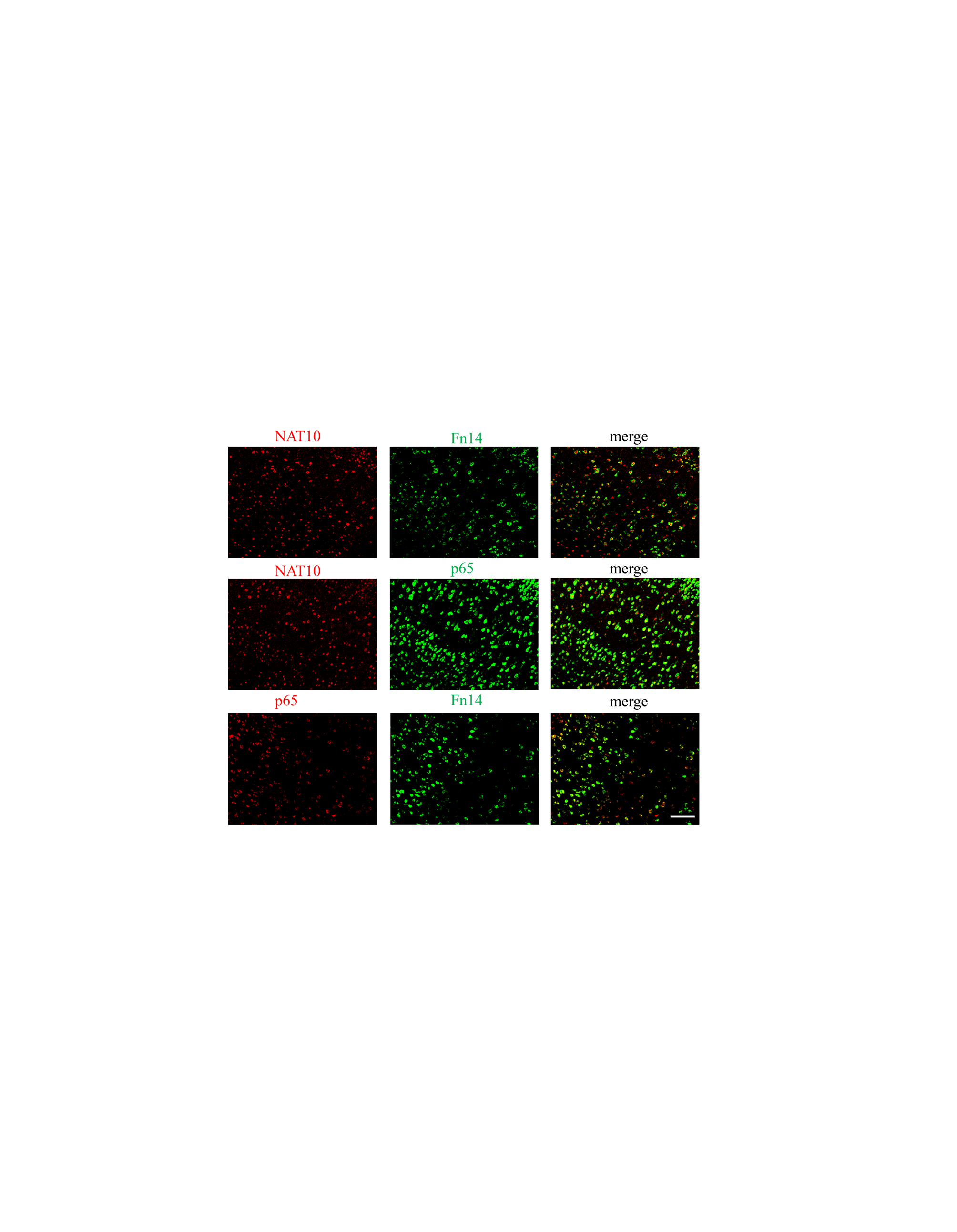

Supplement: Supplementary file 17 — Coexpression of NAT10 with Fn14 or p65 and coexpression of p65 with Fn14. Double immunohistochemical staining revealed that NAT10 immunoreactivity overlapped with Fn14 and p65 immunoreactivity in the regions adjacent to the core of haemorrhagic lesions in the thalamus on day 5 after Coll IV microinjection. p65 immunoreactivity overlapped with Fn14 immunoreactivity in this region. Representative images from 3 biological repeats (n = 3 mice). Scale bar: 50 μm (PNG 477 kb) [file 12035_2024_4454_Fig16_ESM.png]

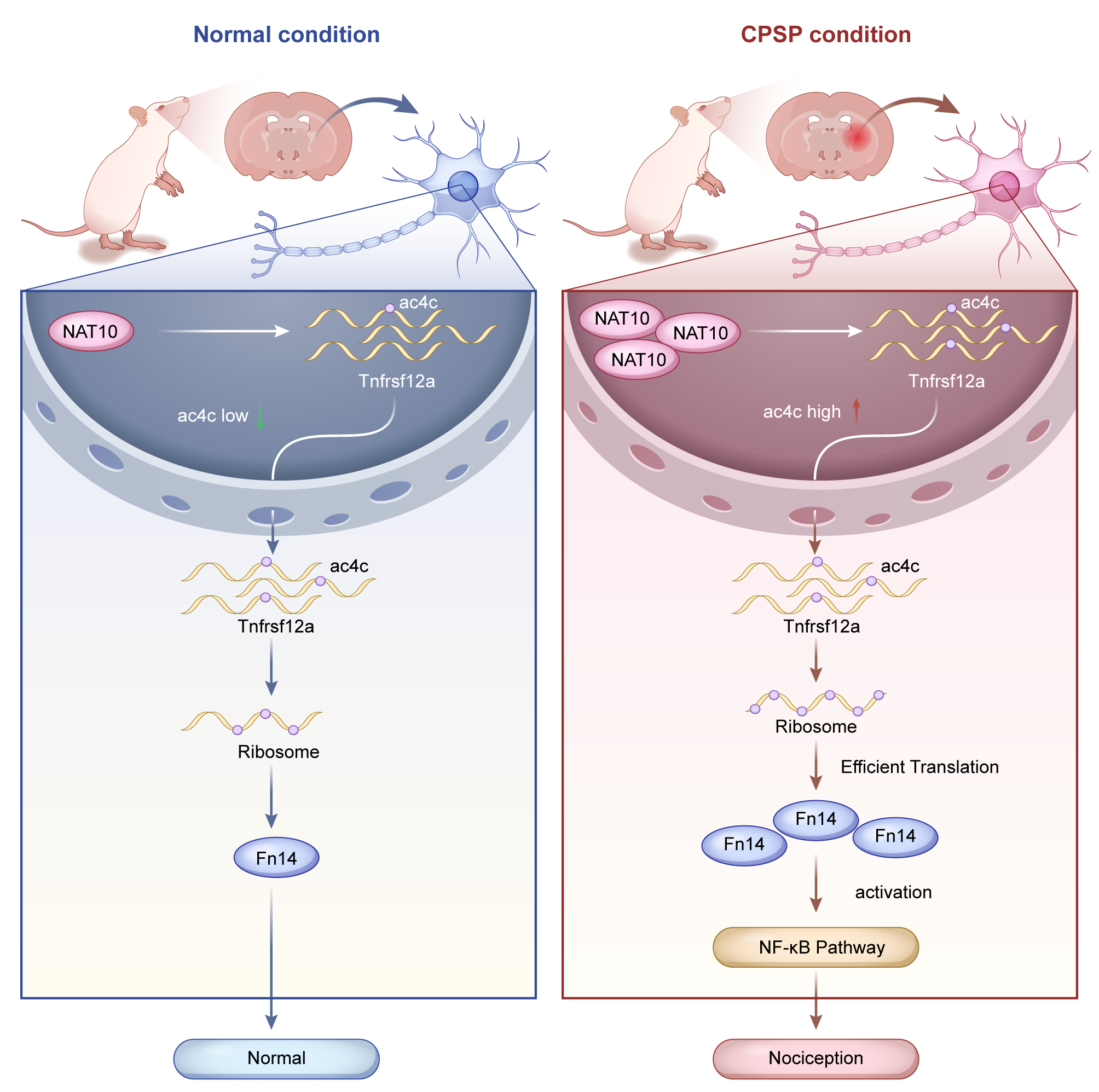

Supplement: Supplementary file 19 — (PNG 501 kb) [file 12035_2024_4454_Fig17_ESM.png]

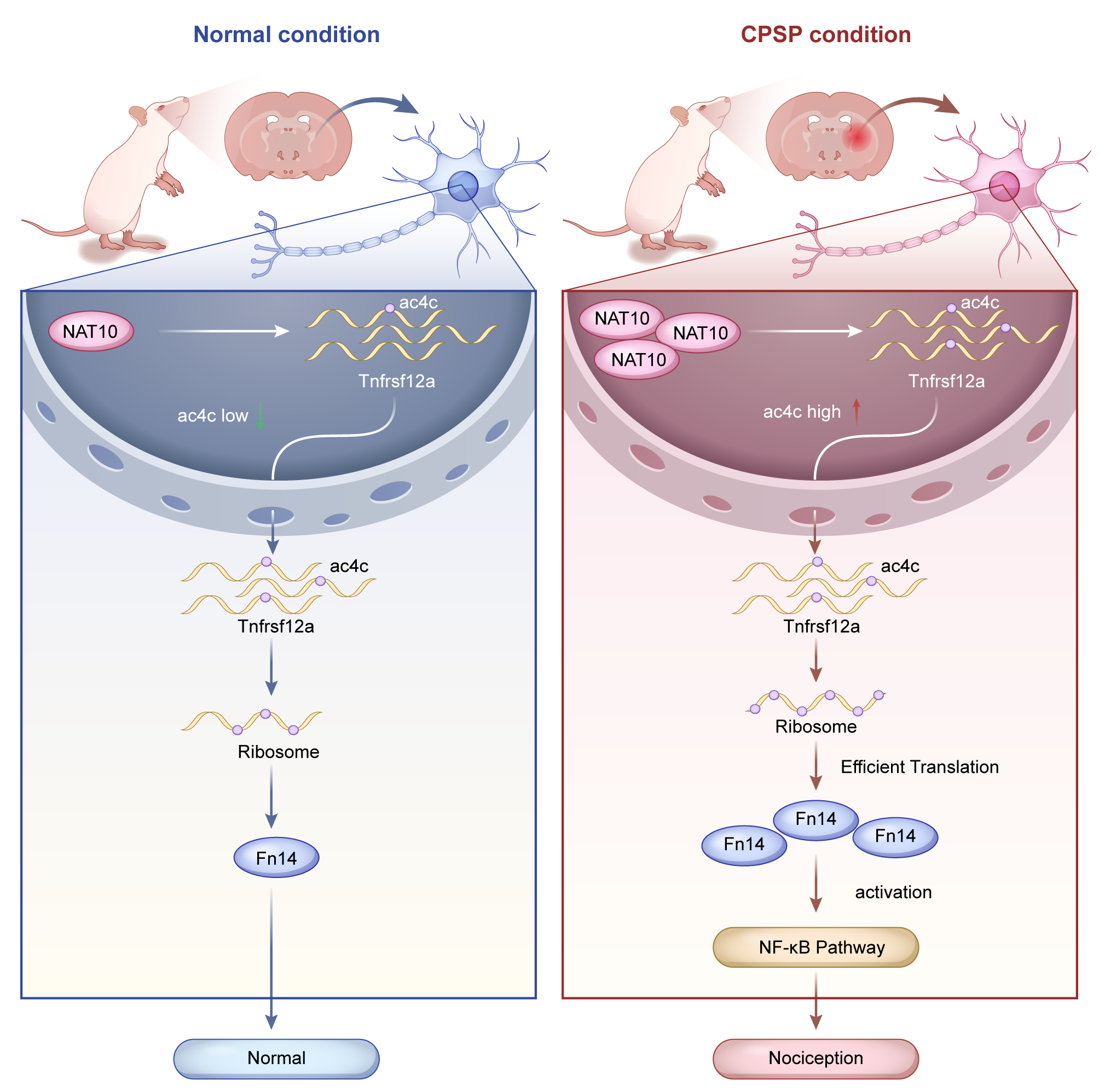

Supplement: Supplementary file 20 — High resolution image (TIF 13362 kb) [file 12035_2024_4454_MOESM10_ESM.tif]
