# Supplementary material for: The Cytidine N-Acetyltransferase NAT10 Promotes Thalamus Hemorrhage-Induced Central Poststroke Pain by Stabilizing Fn14 Expression in Thalamic Neurons
Source: Mol Neurobiol. 2024 Sep 13;62(3):3276–92. doi: 10.1007/s12035-024-04454-4 (PMC11790786; doi:10.1007/s12035-024-04454-4)
Supplement: Supplementary file 21 — (DOC 62 kb) [file 12035_2024_4454_MOESM11_ESM.doc]

**Supplementary Table 1.** Sequences of shRNA and the control.

| Name | Sequences |
| --- | --- |
| shRNA-1 | ACCGTTGCTGTTCACCCAGATTATCCTCGAGGATAATCTGGGTGAACAGCAATTTTTTG |
| shRNA-2 | ACCGAGAGTGGGACCTTGAACTTAACTCGAGTTAAGTTCAAGGTCCCACTCTTTTTTTG |
| shRNA-3 | ACCGGCAGTGGAGAAGTGGCTTAATCTCGAGATTAAGCCACTTCTCCACTGCTTTTTTG |
| scramble (NC) | CTAGCAAAAAACCTAAGGTTAAGTCGCCCTCGCTCGAGCGAGGGCGACTTAACCTTAGG |

Supplementary Table 2. Antibodies for Western blotting.

| Name | Concentration | Company | Catalogue |
| --- | --- | --- | --- |
| rabbit anti-NAT10 | 1:2000 | Abcam | ab194297 |
| rabbit anti-Fn14 | 1:1000 | Cell Signaling Technology | 4403S |
| rabbit anti-p65 | 1:1000 | Cell Signaling Technology | 8242S |
| rabbit anti-GAPDH | 1:1000 | Cell Signaling Technology | 5174S |
| rabbit anti-β-actin | 1:1000 | Cell Signaling Technology | 8457S |
| rabbit anti-histone H3 | 1:1000 | Cell Signaling Technology | 4909S |
| goat anti-rabbit IgG | 1:5000 | Cell Signaling Technology | 7074P2 |

**Supplementary Table 3.** Antibodies for immunofluorescence.

| Name | Concentration | Company | Catalogue |
| --- | --- | --- | --- |
| mouse anti-NAT10 | 1:50 | Santa Cruz Biotechnology | sc-271770 |
| rabbit anti-NeuN | 1:500 | Abcam | ab177487 |
| rabbit anti-GFAP | 1:500 | Abcam | ab7260 |
| rabbit anti-Iba1 | 1:1000 | FUJIFILM Wako Chemicals | 019-19741 |
| rabbit anti-CD68 | 1:800 | Abcam | ab213363 |
| rabbit anti-Fn14 | 1:500 | Abcam | ab109365 |
| rabbit anti-NF-κB p65 | 1:500 | CST Inc | 8242S |
| mouse anti-NF-κB p65 | 1:500 | CST Inc | 6956S |
| donkey anti-mouse IgG conjugated with Cy3 | 1:500 | Jackson  ImmunoResearch Labs | 715-165-150 |
| donkey anti-rabbit IgG conjugated with Cy2 | 1:500 | Jackson ImmunoResearch  Labs | 711-225-152 |
| goat anti-mouse IgG conjugated  with Cy3 | 1:500 | Jackson ImmunoResearch  Labs | 115-165-003 |

**Supplementary Table 4.** Locomotor function.

| Treatment group | Placing | Grasping | Righting |
| --- | --- | --- | --- |
| saline + vehicle | 5 (0) | 5 (0) | 5 (0) |
| Coll IV + vehicle | 5 (0) | 5 (0) | 5 (0) |
| Coll IV + Remodelin 1 mg/kg | 5 (0) | 5 (0) | 5 (0) |
| Coll IV + Remodelin 5 mg/kg | 5 (0) | 5 (0) | 5 (0) |
| Coll IV + Remodelin 10 mg/kg | 5 (0) | 5 (0) | 5 (0) |
| saline + Remodelin | 5 (0) | 5 (0) | 5 (0) |
| saline + vehicle | 5 (0) | 5 (0) | 5 (0) |
| blood + vehicle | 5 (0) | 5 (0) | 5 (0) |
| blood + Remodelin | 5 (0) | 5 (0) | 5 (0) |
| saline + Remodelin | 5 (0) | 5 (0) | 5 (0) |
| saline + NAT10^+/+^ | 5 (0) | 5 (0) | 5 (0) |
| Coll IV + NAT10^+/+^ | 5 (0) | 5 (0) | 5 (0) |
| Coll IV + NAT10^+/-^ | 5 (0) | 5 (0) | 5 (0) |
| saline + NAT10^+/-^ | 5 (0) | 5 (0) | 5 (0) |
| saline + AAV-NC | 5 (0) | 5 (0) | 5 (0) |
| blood + AAV-NC | 5 (0) | 5 (0) | 5 (0) |
| blood + AAV-shRNA | 5 (0) | 5 (0) | 5 (0) |
| saline + AAV-shRNA | 5 (0) | 5 (0) | 5 (0) |
| AAV-NAT10 | 5 (0) | 5 (0) | 5 (0) |
| AAV-EGFP | 5 (0) | 5 (0) | 5 (0) |

NC: negative control shRNA.

n = 8 mice per group; 5 trials; mean (SD).
